# Supplementary material for: TNFPred: identifying tumor necrosis factors using hybrid features based on word embeddings
Source: BMC Med Genomics. 2020 Oct 22;13(Suppl 10):155. doi: 10.1186/s12920-020-00779-w (PMC7579990; doi:10.1186/s12920-020-00779-w)
Supplement: Supplementary file 1 — Additional file 1 Statistical test results. Table S1: F and F-critical values from F-test to test the null hypothesis that two datasets’ variances are equal in terms of single amino acid, dipeptide and tripeptide composition. Table S2: p-values from the unpaired student T-test on single amino acid composition on positive group (18 TNF sequences) and negative group (non-TNF sequences) with the assumption that the variance are equal. Table S3: Top 20 dipeptides with the lowest p-values from the unpaired student T-test on dipeptide composition on positive group (18 TNF sequences) and negative group (non-TNF sequences) with the assumption that the variance are equal. Table S4: Top 20 tripeptides with the lowest p-values from the unpaired student T-test on tripeptide composition on positive group (18 TNF sequences) and negative group (non-TNF sequences) with the assumption that the variance are equal. [file 12920_2020_779_MOESM1_ESM.docx]

# Additional file 1: Statistical test results

This additional file describe the statistical tests on the entire dataset to evaluate the distinction between TNFs and non-TNFs amino acid composition variance

**Table S1**: F and F-critical values from F-test to test the null hypothesis that two datasets’ variances are equal in terms of single amino acid, dipeptide and tripeptide composition

|  | Amino acid | Dipeptide | Tripeptide |
| --- | --- | --- | --- |
| F | 1.063 | 0.489 | 0.892 |
| F Critical one-tail | 2.173 | 0.848 | 0.964 |

**Table S2**: p-values from the unpaired student T-test on single amino acid composition on positive group (18 TNF sequences) and negative group (non-TNF sequences) with the assumption that the variance are equal

| Amino acid | A | R | N | D | C | Q | E | G | H | I |
| --- | --- | --- | --- | --- | --- | --- | --- | --- | --- | --- |
| p-value | 0.434 | 0.229 | 0.402 | 0.410 | 0.008 | 0.289 | 0.414 | 0.005 | 0.492 | 0.124 |
| Amino acid | L | K | M | F | P | S | T | W | Y | V |
| p-value | 0.318 | 0.038 | 0.093 | 0.379 | 0.439 | 0.300 | 0.211 | 0.029 | 0.005 | 0.034 |

**Table S3**: Top 20 dipeptides with the lowest p-values from the unpaired student T-test on dipeptide composition on positive group (18 TNF sequences) and negative group (non-TNF sequences) with the assumption that the variance are equal

| Dipeptide | GL | LY | FG | SW | LV | EG | VY |
| --- | --- | --- | --- | --- | --- | --- | --- |
| p-value | 1.98E-08 | 1.07E-07 | 1.75E-07 | 3.29E-06 | 2.76E-05 | 2.76E-05 | 3.94E-05 |
| Dipeptide | QV | PW | QD | YF | GA | IY | FQ |
| p-value | 4.34E-05 | 6.59E-05 | 7.66E-05 | 3.60E-04 | 3.90E-04 | 6.24E-04 | 7.27E-04 |
| Dipeptide | HL | WE | YW | YY | AG | QI |  |
| p-value | 1.01E-03 | 1.03E-03 | 1.56E-03 | 1.86E-03 | 2.11E-03 | 2.35E-03 |  |

**Table S4**: Top 20 tripeptides with the lowest p-values from the unpaired student T-test on tripeptide composition on positive group (18 TNF sequences) and negative group (non-TNF sequences) with the assumption that the variance are equal

| Tripeptide | GLY | QDG | TFF | FFG | FGA | LYY | VYV |
| --- | --- | --- | --- | --- | --- | --- | --- |
| p-value | 5.22E-27 | 2.15E-16 | 1.05E-13 | 2.02E-12 | 1.35E-09 | 1.35E-09 | 3.88E-09 |
| Tripeptide | YYL | YSQ | RNS | HLT | YLY | NMP | QNG |
| p-value | 3.88E-09 | 3.9E-09 | 4.04E-07 | 4.04E-07 | 4.04E-07 | 4.58E-07 | 4.58E-07 |
| Tripeptide | HPD | IYS | MKG | YYV | RRG | DGL |  |
| p-value | 4.58E-07 | 4.58E-07 | 4.58E-07 | 4.58E-07 | 6.02E-07 | 6.25E-07 |  |
