# Supplementary material for: TNFPred: identifying tumor necrosis factors using hybrid features based on word embeddings
Source: BMC Med Genomics. 2020 Oct 22;13(Suppl 10):155. doi: 10.1186/s12920-020-00779-w (PMC7579990; doi:10.1186/s12920-020-00779-w)
Supplement: Supplementary file 2 — Additional file 2 Visualization of feature sets. Fig. S1a: Visualization corresponding to protein feature vectors comprised from 1-g embedding vectors with perplexity equal to 25. Fig. S1b: Visualization corresponding to protein feature vectors comprised from 1-g embedding vectors with perplexity equal to 50. Fig. S2a: Visualization corresponding to protein feature vectors comprised from 2-g embedding vectors with perplexity equal to 25. Fig. S2b: Visualization corresponding to protein feature vectors comprised from 2-g embedding vectors with perplexity equal to 50. Fig. S3a: Visualization corresponding to protein feature vectors comprised from 3-g embedding vectors with perplexity equal to 25. Fig. S3b: Visualization corresponding to protein feature vectors comprised from 3-g embedding vectors with perplexity equal to 50. Fig. S4a: Visualization corresponding to protein feature vectors comprised from 4-g embedding vectors with perplexity equal to 25. Fig. S4b: Visualization corresponding to protein feature vectors comprised from 4-g embedding vectors with perplexity equal to 50. Fig. S5a: Visualization corresponding to protein feature vectors comprised from 5-g embedding vectors with perplexity equal to 25. Fig. S5b: Visualization corresponding to protein feature vectors comprised from 5-g embedding vectors with perplexity equal to 50. Fig. S6a: Visualization corresponding to protein feature vectors comprised from 1-g and 2-g embedding combined vectors with perplexity equal to 25. Fig. S6b: Visualization corresponding to protein feature vectors comprised from 1-g and 2-g embedding combined vectors with perplexity equal to 50. Fig. S7a: Visualization corresponding to protein feature vectors comprised from 1-g and 3-g embedding combined vectors with perplexity equal to 25. Fig. S7b: Visualization corresponding to protein feature vectors comprised from the combination of 1-g and 3-g embedding combined vectors with perplexity equal to 50. Fig. S8a: Visualization corres [file 12920_2020_779_MOESM2_ESM.docx]

# Additional file 2: Visualization of feature sets

This additional file displays the visualization of feature sets using t-Distributed Stochastic Neighbor Embedding

| Perplexity=25 | Perplexity = 50 |
| --- | --- |
| 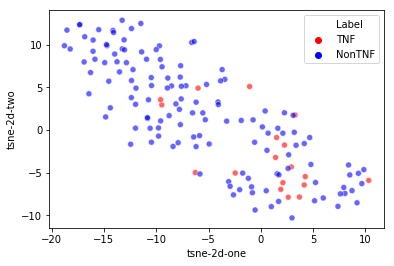  Figure S1a: Visualization corresponding to protein feature vectors comprised from 1-gram embedding vectors with perplexity equal to 25 | 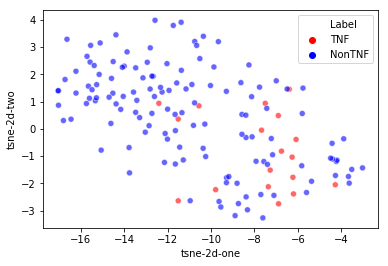  Figure S1b: Visualization corresponding to protein feature vectors comprised from 1-gram embedding vectors with perplexity equal to 50 |
|  |  |
| 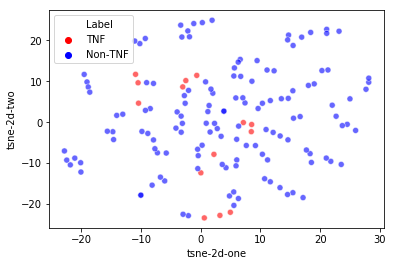Figure S2a: Visualization corresponding to protein feature vectors comprised from 2-gram embedding vectors with perplexity equal to 25 | 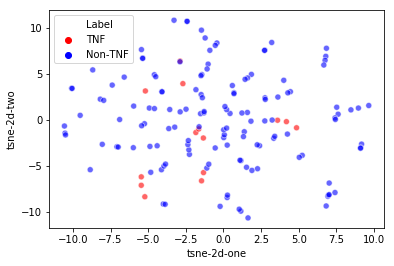Figure S2b: Visualization corresponding to protein feature vectors comprised from 2-gram embedding vectors with perplexity equal to 50 |
| 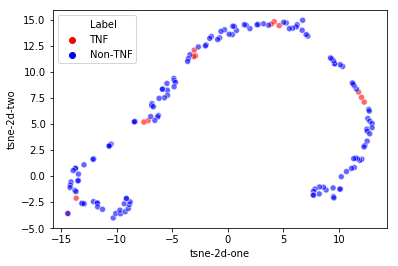  Figure S3a: Visualization corresponding to protein feature vectors comprised from 3-gram embedding vectors with perplexity equal to 25 | 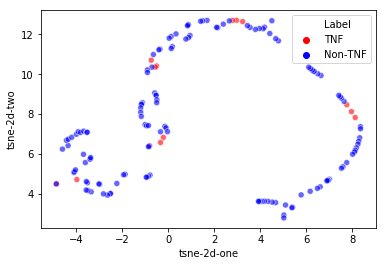  Figure S3b: Visualization corresponding to protein feature vectors comprised from 3-gram embedding vectors with perplexity equal to 50 |
| 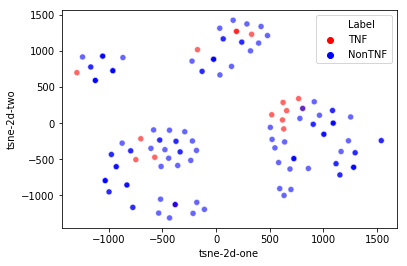  Figure S4a: Visualization corresponding to protein feature vectors comprised from 4-gram embedding vectors with perplexity equal to 25 | 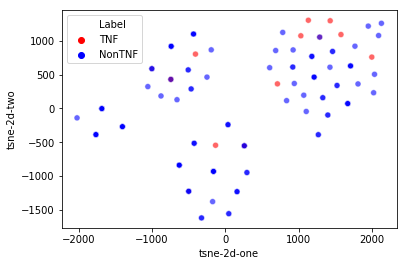Figure S4b: Visualization corresponding to protein feature vectors comprised from 4-gram embedding vectors with perplexity equal to 50 |
| 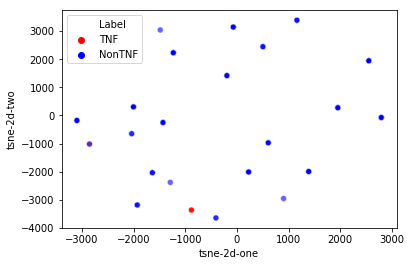  Figure S5a: Visualization corresponding to protein feature vectors comprised from 5-gram embedding vectors with perplexity equal to 25 | 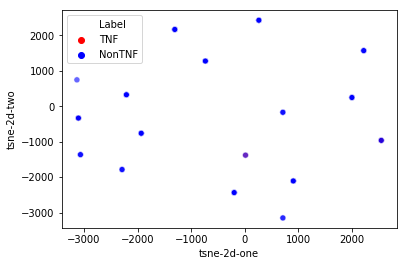Figure S5b: Visualization corresponding to protein feature vectors comprised from 5-gram embedding vectors with perplexity equal to 50 |
| 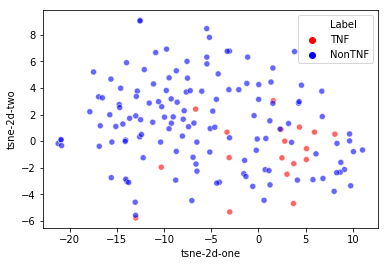  Figure S6a: Visualization corresponding to protein feature vectors comprised from 1-gram and 2-gram embedding combined vectors with perplexity equal to 25 | 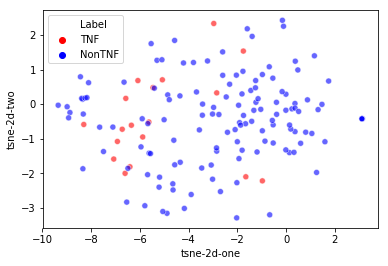Figure S6b: Visualization corresponding to protein feature vectors comprised from 1-gram and 2-gram embedding combined vectors with perplexity equal to 50 |
| 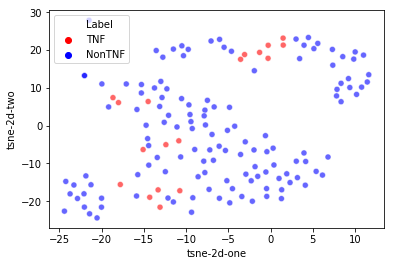  Figure S7a: Visualization corresponding to protein feature vectors comprised from 1-gram and 3-gram embedding combined vectors with perplexity equal to 25 | 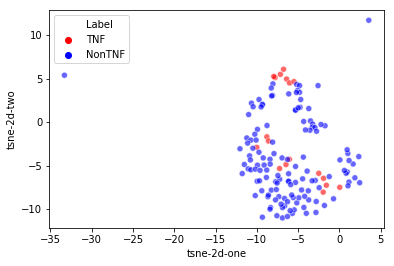  Figure S7b: Visualization corresponding to protein feature vectors comprised from the combination of 1-gram and 3-gram embedding combined vectors with perplexity equal to 50 |
| 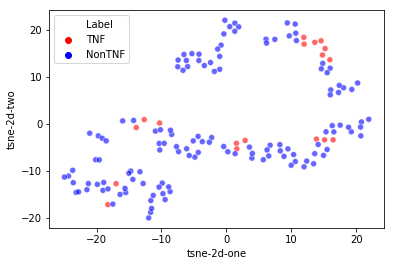  Figure S8a: Visualization corresponding to protein feature vectors comprised from 2-gram and 3-gram embedding combined vectors with perplexity equal to 25 | 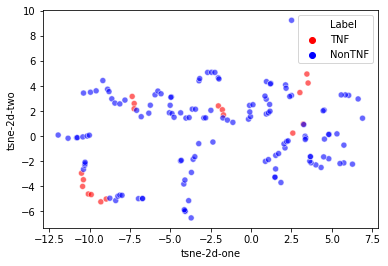  Figure S8b: Visualization corresponding to protein feature vectors comprised from the combination of 2-gram and 3-gram embedding combined vectors with perplexity equal to 50 |
