# Supplementary material for: TNFPred: identifying tumor necrosis factors using hybrid features based on word embeddings
Source: BMC Med Genomics. 2020 Oct 22;13(Suppl 10):155. doi: 10.1186/s12920-020-00779-w (PMC7579990; doi:10.1186/s12920-020-00779-w)
Supplement: Supplementary file 3 — Additional file 3 The details of the feature extraction method with examples. [file 12920_2020_779_MOESM3_ESM.docx]

**Additional file 3: The details of the feature extraction method with examples**

This additional file provides a step-by-step demonstration of our feature extraction method with 3 sample sequences and selected n-gram length of 3

| Sequence | Sequence content | Overlapping n-grams |
| --- | --- | --- |
| Seq1 | M**TAAS**QANPYSI | - MTA **TAA AAS** ASQ SQA QAN ANP NPY PYS YSI (10 trigrams) |
| Seq2 | D**TAAS**VTMSGAKGF | - DTA **TAA AAS** ASV SVT VTM TMS MSG SGA GAK AKG KGF (12 trigrams) |
| Seq3 | GNGKVHTQ**TAASN**RFV**AAS** | - GNG NGK GKV KVH VHT HTQ **TAA AAS ASN** NRF RFV VAA **AAS** (13 trigrams) |

Split sequence into overlapping n-grams

1

2

MTA^1^ **TAA^2^ AAS^3^** ASQ^4^ SQA^5^ QAN^6^ ANP^7^ NPY^8^ PYS^9^ YSI^10^ DTA^11^ ASV^12^ SVT^13^ VTM^14^ TMS^15^ MSG^16^ SGA^17^ GAK^18^ AKG^19^ KGF^20^ GNG^21^ NGK^22^ GKV^23^ KVH^24^ VHT^25^ HTQ^26^ ASN^27^ NRF^28^ RFV^29^ VAA^30^

**Note: superscriptted number: index of ngram in the vocabulary*

Building the vocabulary

3

Using FastText to create (n-gram: one-dimension embedding vector) pairs

| No. | (Ngram🡪Embedding vector) pair  value vector | | No. | (Ngram🡪Embedding vector) pair | | No. | (Ngram🡪Embedding vector) pair | |
| --- | --- | --- | --- | --- | --- | --- | --- | --- |
| 1 | MTA🡪 | (0.9891) | 11 | DTA🡪 | (0.9546) | 21 | GNG🡪 | (1.004) |
| 2 | TAA🡪 | (1.0671) | 12 | ASV🡪 | (0.8894) | 22 | NGK🡪 | (0.9214) |
| 3 | AAS🡪 | (1.0654) | 13 | SVT🡪 | (0.8828) | 23 | GKV🡪 | (0.9581) |
| 4 | ASQ🡪 | (1.0096) | 14 | VTM🡪 | (0.8964) | 24 | KVH🡪 | (0.8148) |
| 5 | SQA🡪 | (1.0147) | 15 | TMS🡪 | (1.0257) | 25 | VHT🡪 | (0.9581) |
| 6 | QAN🡪 | (0.9790) | 16 | MSG🡪 | (0.8317) | 26 | HTQ🡪 | (0.9097) |
| 7 | ANP🡪 | (1.0337) | 17 | SGA🡪 | (0.9261) | 27 | ASN🡪 | (0.9905) |
| 8 | NPY🡪 | (0.8685) | 18 | GAK🡪 | (0.9033) | 28 | NRF🡪 | ( 0.9155) |
| 9 | PYS🡪 | (0.9546) | 19 | AKG🡪 | (0.8597) | 29 | RFV🡪 | ( 0.9113) |
| 10 | YSI🡪 | (0.8774) | 20 | KGF🡪 | (0.9331) | 30 | VAA🡪 | (0.8989) |

| Position in vocabulary | 1 | 2 | 3 | 4 | 5 | 6 | 7 | 8 | 9 | 10 | 11 | 12 | 13 | 14 | 15 | 16 | 17 | 18 | 19 | 20 | 21 | 22 | 23 | 24 | 25 | 26 | 27 | 28 | 29 | 30 |
| --- | --- | --- | --- | --- | --- | --- | --- | --- | --- | --- | --- | --- | --- | --- | --- | --- | --- | --- | --- | --- | --- | --- | --- | --- | --- | --- | --- | --- | --- | --- |
| 3-gram | MTA | TAA | AAS | ASQ | SQA | QAN | ANP | NPY | PYS | YSI | DTA | ASV | SVT | VTM | TMS | MSG | SGA | GAK | AKG | KGF | GNG | NGK | GKV | KVH | VHT | HTQ | ASN | NRF | RFV | VAA |
| Frequency of 3-grams in Seq1 | 1 | 1 | 1 | 1 | 1 | 1 | 1 | 1 | 1 | 1 | 0 | 0 | 0 | 0 | 0 | 0 | 0 | 0 | 0 | 0 | 0 | 0 | 0 | 0 | 0 | 0 | 0 | 0 | 0 | 0 |
| Frequency of 3-grams in Seq2 | 0 | 0 | 0 | 0 | 0 | 0 | 0 | 0 | 0 | 0 | 1 | 1 | 1 | 1 | 1 | 1 | 1 | 1 | 1 | 1 | 1 | 1 | 0 | 0 | 0 | 0 | 0 | 0 | 0 | 0 |
| Frequency of 3-grams in Seq3 | 0 | 1 | 2 | 0 | 0 | 0 | 0 | 0 | 0 | 0 | 0 | 0 | 0 | 0 | 0 | 0 | 0 | 0 | 0 | 0 | 1 | 1 | 1 | 1 | 1 | 1 | 1 | 1 | 1 | 1 |

Converting each protein sequence into fix-sized vector based on frequency of motifs

4

The feature vector of each sequence is calculated as:

i^th^ feature = (frequency at i^th^ position) x (embedding vector)

In our study, the dimension of the embedding vector is equal to 1, therefore the embedding vector is a scalar value.

Finally, the word embedding-based feature vectors are as below:

**seq1:** (0.9891, 1.0671, 1.0654, 1.0096, 1.0147, 0.9791, 1.0337, 0.8685, 0.9546, 0.8775, 0, 0, 0, 0, 0, 0, 0, 0, 0, 0, 0, 0, 0, 0, 0, 0, 0, 0, 0, 0)

**seq2:** (0, 0, 0, 0, 0, 0, 0, 0, 0, 0, 0.9546, 1.0671, 1.0654, 0.8895, 0.8829, 0.8965, 1.0257, 0.8318, 0.9262, 0.9034, 0.8598, 0.9332, 0, 0, 0, 0, 0, 0, 0, 0)

**seq3:** (0, 1.0671, 2.1308, 0, 0, 0, 0, 0, 0, 0, 0, 0, 0, 0, 0, 0, 0, 0, 0, 0, 1.0040, 0.9214, 0.9582, 0.8149, 0.9581, 0.9098, 0.9905, 0.9155, 0.9114, 0.8989)

These vectors were the input of binary classifiers.
