# Supplementary material for: TNFPred: identifying tumor necrosis factors using hybrid features based on word embeddings
Source: BMC Med Genomics. 2020 Oct 22;13(Suppl 10):155. doi: 10.1186/s12920-020-00779-w (PMC7579990; doi:10.1186/s12920-020-00779-w)
Supplement: Supplementary file 4 — Additional file 4 Formulae of assessment metrics. [file 12920_2020_779_MOESM4_ESM.docx]

# Additional file 4: Formulae of assessment metrics

This additional file describe the formulae of assessment metrics to evaluate the prediction performance

$$Sen=1-\frac{N_{-}^{+}}{N^{+}} , 0\leq Sen\leq1 (1)$$

$$Spec=1-\frac{N_{+}^{-}}{N^{-}} , 0\leq Spec\leq1 (2)$$

$$Acc=1-\frac{N_{-}^{+}+N_{+}^{-}}{N^{+}+N^{-}} , 0\leq Acc\leq1 (3)$$

$MCC= \frac{1-\left( \frac{N_{-}^{+}}{N^{+}}+\frac{N_{+}^{-}}{N^{-}} \right)}{\sqrt{\left( 1+\frac{N_{+}^{-}-N_{-}^{+}}{N^{+}} \right)\left( 1+\frac{N_{-}^{+}-N_{+}^{-}}{N^{-}} \right)}} , -1\leq MCC\leq1 (4)$

TP, FP, TN, FN stand for true positive, false positive, true negative, and false negative respectively.

The relations between these symbols and the symbols in Eqs. (1, 2, 3 and 4) are given by:

$$\left\{ \begin{matrix} N_{+}^{-}=FP \\ N_{-}^{+}=FN \\ \begin{matrix} N^{+}=TP+N_{-}^{+} \\ N^{-}=TN+N_{+}^{-} \end{matrix} \end{matrix} \right. \left( 5 \right)$$

where, $N_{-}^{+}$ represents the number of positive samples (TNFs) incorrectly predicted to be negative samples (non-TNFs), $N_{+}^{-}$ represents the number of negative samples incorrectly predicted to be positive sample, $N^{+}$represents the number of positive sample surveyed and $N^{-}$represents the number of negative sample surveyed.
