# Supplementary material for: TNFPred: identifying tumor necrosis factors using hybrid features based on word embeddings
Source: BMC Med Genomics. 2020 Oct 22;13(Suppl 10):155. doi: 10.1186/s12920-020-00779-w (PMC7579990; doi:10.1186/s12920-020-00779-w)
Supplement: Supplementary file 5 — Additional file 5 The surveyed dataset. [file 12920_2020_779_MOESM5_ESM.docx]

**Additional file 5: The surveyed dataset**

This additional file provides the protein sequences of data used in this study

The amino acid sequence of 151 surveyed proteins belong to 6 families (TNF, chemokine, interferon, interleukin, TGF-beta and others) are given below with the first line indicating the protein ID.

*****TNF**

>O54907

MAARRSQRRRGRRGEPGTALLAPLVLSLGLALACLGLLLVVVSLGSWATLSAQEPSQEELTAEDRREPPELNPQTEESQDVVPFLEQLVRPRRSAPKGRKARPRRAIAAHYEVHPRPGQDGAQAGVDGTVSGWEETKINSSSPLRYDRQIGEFTVIRAGLYYLYCQVHFDEGKAVYLKLDLLVNGVLALRCLEEFSATAASSPGPQLRLCQVSGLLPLRPGSSLRIRTLPWAHLKAAPFLTYFGLFQVH

>O55237

MPEEGRPCPWVRWSGTAFQRQWPWLLLVVFITVFCCWFHCSGLLSKQQQRLLEHPEPHTAELQLNLTVPRKDPTLRWGAGPALGRSFTHGPELEEGHLRIHQDGLYRLHIQVTLANCSSPGSTLQHRATLAVGICSPAAHGISLLRGRFGQDCTVALQRLTYLVHGDVLCTNLTLPLLPSRNADETFFGVQWICP

>O75888

MPASSPFLLAPKGPPGNMGGPVREPALSVALWLSWGAALGAVACAMALLTQQTELQSLRREVSRLQGTGGPSQNGEGYPWQSLPEQSSDALEAWENGERSRKRRAVLTQKQKKQHSVLHLVPINATSKDDSDVTEVMWQPALRRGRGLQAQGYGVRIQDAGVYLLYSQVLFQDVTFTMGQVVSREGQGRQETLFRCIRSMPSHPDRAYNSCYSAGVFHLHQGDILSVIIPRARAKLNLSPHGTFLGFVKL

>P32972

MEPGLQQAGSCGAPSPDPAMQVQPGSVASPWRSTRPWRSTSRSYFYLSTTALVCLVVAVAIILVLVVQKKDSTPNTTEKAPLKGGNCSEDLFCTLKSTPSKKSWAYLQVSKHLNNTKLSWNEDGTIHGLIYQDGNLIVQFPGLYFIVCQLQFLVQCSNHSVDLTLQLLINSKIKKQTLVTVCESGVQSKNIYQNLSQFLLHYLQVNSTISVRVDNFQYVDTNTFPLDNVLSVFLYSSSD

>P41273

MEYASDASLDPEAPWPPAPRARACRVLPWALVAGLLLLLLLAAACAVFLACPWAVSGARASPGSAASPRLREGPELSPDDPAGLLDLRQGMFAQLVAQNVLLIDGPLSWYSDPGLAGVSLTGGLSYKEDTKELVVAKAGVYYVFFQLELRRVVAGEGSGSVSLALHLQPLRSAAGAAALALTVDLPPASSEARNSAFGFQGRLLHLSAGQRLGVHLHTEARARHAWQLTQGATVLGLFRVTPEIPAGLPSPRSE

>P41274

MDQHTLDVEDTADARHPAGTSCPSDAALLRDTGLLADAALLSDTVRPTNAALPTDAAYPAVNVRDREAAWPPALNFCSRHPKLYGLVALVLLLLIAACVPIFTRTEPRPALTITTSPNLGTRENNADQVTPVSHIGCPNTTQQGSPVFAKLLAKNQASLCNTTLNWHSQDGAGSSYLSQGLRYEEDKKELVVDSPGLYYVFLELKLSPTFTNTGHKVQGWVSLVLQAKPQVDDFDNLALTVELFPCSMENKLVDRSWSQLLLLKAGHRLSVGLRAYLHGAQDAYRDWELSYPNTTSFGLFLVKPDNPWE

>P50592

MPSSGALKDLSFSQHFRMMVICIVLLQVLLQAVSVAVTYMYFTNEMKQLQDNYSKIGLACFSKTDEDFWDSTDGEILNRPCLQVKRQLYQLIEEVTLRTFQDTISTVPEKQLSTPPLPRGGRPQKVAAHITGITRRSNSALIPISKDGKTLGQKIESWESSRKGHSFLNHVLFRNGELVIEQEGLYYIYSQTYFRFQEAEDASKMVSKDKVRTKQLVQYIYKYTSYPDPIVLMKSARNSCWSRDAEYGLYSIYQGGLFELKKNDRIFVSVTNEHLMDLDQEASFFGAFLIN

>Q5UBV8

MAEELGLGFGEGVPVEVLPEGCRHRPEARAGLAARSKACLALTCCLLSFPILAGLSTLLMAGQLRVPGKDCMLRAITEERSEPSPQQVYSPPRGKPRAHLTIKKQTPAPHLKNQLSALHWEHDLGMAFTKNGMKYINKSLVIPESGDYFIYSQITFRGTTSVCGDISRGRRPNKPDSITVVITKVADSYPEPARLLTGSKSVCEISNNWFQSLYLGAMFSLEEGDRLMVNVSDISLVDYTKEDKTFFGAFLL

>Q7TS55

MEEMPLRESSPQRAERCKKSWLLCIVALLLMLLCSLGTLIYTSLKPTAIESCMVKFELSSSKWHMTSPKPHCVNTTSDGKLKILQSGTYLIYGQVIPVDKKYIKDNAPFVVQIYKKNDVLQTLMNDFQILPIGGVYELHAGDNIYLKFNSKDHIQKTNTYWGIILMPDLPFIS

>Q8JFG3

MGAYTTAPCDLEMGPEERTVVLIEKKSSTGWMWKVSVALLIAALCFAGVLLFAWYWNGKPEILIHSGQSEALTKKDHAEKTDPHSTLKRISSKAKAAIHLEGSYDEDEGLKDQVEWKNGQGQAFAQGGFRLVDNKIVIPHTGLYFVYSQASFRVSCSDGDEEGAGRHLTPLSHRISRYSESMGSDVSLMSAVRSACQNTAQEDSYSDGRGWYNTIYLGAVFQLNRGDKLETETNQLSELETDEGKTFFGVFAL

>Q8MUJ1

MTAETLKPFITPTSANDDGFPAKATSTATAQRRTRQLIPLVLGFIGLGLVVAILALTIWQTTRVSHLDKELKSLKRVVDNLQQRLGINYLDEFDEFQKEYENALIDYPKKVDGLTDEEDDDDGDGLDSIADDEDDDVSYSSVDDVGADYEDYTDMLNKLNNAHTGTTPTSETTAEGEGETDSASSASNDDNVFDDFTSYNAHKKKQERKSRSIADVRNEEQNIQGNHTELQEKSSNEATSKESPAPLHHRRRMHSRHRHLLVRKGESLLSARSEDSRPAAHFHLSSRRRHQGSMGYHGDMYIGNDNERNSYQGHFQTRDGVLTVTNTGLYYVYAQICYNNSHDQNGFIVFQGDTPFLQCLNTVPTNMPHKVHTCHTSGLIHLERNERIHLKDIHNDRNAVLREGNNRSYFGIFKV

>Q9BEA8

MQQPFNYPYPQIFWVDSSATSPWASPGSVFPCPASVPGRPGQRRPPPPPPPPPPPPTLLPSRPLPPLPPPSLKKKRDHNAGLCLLVMFFMVLVALVGLGLGMFQLFHLQKELTELRESASQRHTESSLEKQIGHPNLPSEKKELRKVAHLTGKPNSRSIPLEWEDTYGIALVSGVKYMKGSLVINDTGLYFVYSKVYFRGQYCNNQPLSHKVYTRNSRYPQDLVLMEGKMMNYCTTGQMWARSSYLGAVFNLTSADHLYVNVSELSLVNFEESKTFFGLYKL

>Q9ESE2

MRRANRDYGKYLRGSEEMGSCPGVPHEGPLHPAPSAPAPAPPPAASRFMFLALLGLGLGQVVCSIALFLYFRAQMDPNRISEDSTRCFYRILRLRENTGLQDSTLESEDTEALPDSCRRMKQAFQGAVQRELQHIVGPQRFSGVPAMMEGSWLDVARRGKPEAQPFAHLTINAADIPSGSHKVSLSSWYHDRGWAKISNMTLSNGKLRVNQDGFYYLYANICFRHHETSGSVPADYLQLMVYVVKTSIKIPSSHNLMKGGSTKNWSGNSEFHFYSINVGGFFKLRAGEEISVQVSNPSLLDPDQDATYFGAFKVQDID

>Q9JM10

MGALGLQGRGGRPQGTGCLLLAVAGATSLVTLLLAVPITVLAVLALVPQEQGGLVMESAGLGAQAQQGLSKSNGLPSRLHSQIPSSSKNPFLRPGALSSHGKHPWVATLPPIVASTPVPGFQQLQEEKPETDLSSRLPAAHLIGAWMKGQGLSWEAKKEEAFLRSGTQFSGAEGLALPQDGLYYLYCNVGYRGRAPPSGAGPQDRSVTLRSSLYRAGGAYGRGAPELLLEGAETVTPVLDRAGRPQYRPLWYTSVGFGGLVQLRRGERVYVNISHPDMVDYRRGKTFFGAVMVGLVPSASLGKCLHSANV

>Q9TSV8

AWITGQGLGWEAKKEEAFLRSGTQFSGAEGLALPQDGLYYLYCHVGYRGRAPPPGGDPLDRSVTLLSRLYRAGGAYGPGTPELLLEGAETVTPVLDPSRRHEYGPLWYTSVGFGGLVQLRRGERVYVNISHPDMVDYRRGKTFFGAVMVG

>Q9UNG2

MTLHPSPITCEFLFSTALISPKMCLSHLENMPLSHSRTQGAQRSSWKLWLFCSIVMLLFLCSFSWLIFIFLQLETAKEPCMAKFGPLPSKWQMASSEPPCVNKVSDWKLEILQNGLYLIYGQVAPNANYNDVAPFEVRLYKNKDMIQTLTNKSKIQNVGGTYELHVGDTIDLIFNSEHQVLKNNTYWGIILLANPQFIS

>Q9WU72

MDESAKTLPPPCLCFCSEKGEDMKVGYDPITPQKEEGAWFGICRDGRLLAATLLLALLSSSFTAMSLYQLAALQADLMNLRMELQSYRGSATPAAAGAPELTAGVKLLTPAAPRPHNSSRGHRNRRAFQGPEETEQDVDLSAPPAPCLPGCRHSQHDDNGMNLRNIIQDCLQLIADSDTPTIRKGTYTFVPWLLSFKRGNALEEKENKIVVRQTGYFFIYSQVLYTDPIFAMGHVIQRKKVHVFGDELSLVTLFRCIQNMPKTLPNNSCYSAGIARLEEGDEIQLAIPRENAQISRNGDDTFFGALKLL

>Q9Z2P3

MEGEGVQPPDENLENGSRPRFKWKKVLRLVVSGIKAAGLLLCVVYVCLQFSSSPAKDSPIQRLRAPVTGCEGGRLFIGTSKNEYETMEVQNNSVIINCDGLYLIHLKGSFFQEVKINLHFRKDRSPIFVPMLNNGQRVVFTVVTSLAFKDEVYLTVNASDTLCEHLQINDGELIIVQLTPNGYCAPERPYSSTVNQVPL

*****Chemokine**

>B0R191

MKTLAAFLLLSCLIAGEVNGQDRSSRARCFCVDKGLNMVLLKNLDKVEIFPPSPSCNKHEIVVTLKNGAGQKCLNPDSKFTKNVVLKAIGKRMQQSVPHSTTTGTVKSSMTSSTSAPTAFK

>O00585

MAQSLALSLLILVLAFGIPRTQGSDGGAQDCCLKYSQRKIPAKVVRSYRKQEPSLGCSIPAILFLPRKRSQAELCADPKELWVQQLMQHLDKTPSPQKPAQGCRKDRGASKTGKKGKGSKGCKRTERSQTPKGP

>O15467

MKVSEAALSLLVLILIITSASRSQPKVPEWVNTPSTCCLKYYEKVLPRRLVVGYRKALNCHLPAIIFVTKRNREVCTNPNDDWVQEYIKDPNLPLLPTRNLSTVKIITAKNGQPQLLNSQ

>O43927

MKFISTSLLLMLLVSSLSPVQGVLEVYYTSLRCRCVQESSVFIPRRFIDRIQILPRGNGCPRKEIIVWKKNKSIVCVDPQAEWIQRMMEVLRKRSSSTLPVPVFKRKIP

>O70460

MAPRVTPLLAFSLLVLWTFPAPTLGGANDAEDCCLSVTQRPIPGNIVKAFRYLLNEDGCRVPAVVFTTLRGYQLCAPPDQPWVDRIIRRLKKSSAKNKGNSTRRSPVS

>O95715

MSLLPRRAPPVSMRLLAAALLLLLLALYTARVDGSKCKCSRKGPKIRYSDVKKLEMKPKYPHCEEKMVIITTKSVSRYRGQEHCLHPKLQSTKRFIKWYNAWNEKRRVYEE

>P02775

MSLRLDTTPSCNSARPLHALQVLLLLSLLLTALASSTKGQTKRNLAKGKEESLDSDLYAELRCMCIKTTSGIHPKNIQSLEVIGKGTHCNQVEVIATLKDGRKICLDPDAPRIKKIVQKKLAGDESAD

>P10148

MQVPVMLLGLLFTVAGWSIHVLAQPDAVNAPLTCCYSFTSKMIPMSRLESYKRITSSRCPKEAVVFVTKLKREVCADPKKEWVQTYIKNLDRNQMRSEPTTLFKTASALRSSAPLNVKLTRKSEANASTTFSTTTSSTSVGVTSVTVN

>P18340

MKSAVLFLLGIIFLEQCGVRGTLVIRNARCSCISTSRGTIHYKSLKDLKQFAPSPNCNKTEIIATLKNGDQTCLDPDSANVKKLMKEWEKKISQKKKQKRGKKHQKNMKNRKPKTPQSRRRSRKTT

>P21972

MQSNISIYVLTVIGSCFYNPFILTYECRDDCCNGRYGPVPAPWKVLNCTKTGPGCPDSGYLLTTSENKTYCITGNETDKGNYPQTIGAIFPNCSGMNVGAGRLITRMLEEYPRGKSPSNNSINNIKISC

>P21973

MYTRYKLSILFFVINFYNILCMPLSCETECCMAGKKYDDAAIDRDLCVLLCNLQYLASSNEGIGEILQCCLSSNYTSKTREDLRNCIAKCPPLPDRGCTGECCDLRENVDSLRANNPLGCCNDYTKVSSSSLNEDDVIDCRKSDASCEDRGYLLVRNNGSAVCIPENSKNDNIGFYFGSECSDLSRKG

>P30034

QEWSLPGTRVPPPADPEGGDANLRCVCVKTISGVSPKHISSLEVIGAGPHCPSPQLIATLKKGHKICLDPQNLLYKKIIKKLLKSQLLTA

>P30782

ALTELRCQCLQTVQGIHLKNIQNLKVLSPGPHCAQTEVIATLKSGQEACRNPAAPMVKKFLQKRLSNGNSS

>P50228

MSLQLRSSARIPSGSISPFMRMAPLAFLLLFTLPQHLAEAAPSSVIAATELRCVCLTVTPKINPKLIANLEVIPAGPQCPTVEVIAKLKNQKEVCLDPEAPVIKKIIQKILGSDKKKAKRNALAVERTASVQ

>P51670

MKPFHTALSFLILTTALGIWAQITHATETKEVQSSLKAQQGLEIEMFHMGFQDSSDCCLSYNSRIQCSRFIGYFPTSGGCTRPGIIFISKRGFQVCANPSDRRVQRCIERLEQNSQPRTYKQ

>P52460

MAIGFICSSPDAELFSEKSRMSSSVLLGCLLCCMDWSAAVPGKTEPFRKLFDAIMIKKLKSCSAAYPSDLDEGSMCDMADASPTSLELGLSKLDKES

>P82535

GPVAAVVRELRCVCLSTTAIHPKMIARLQVIAAGPQCSKVEVVASLKNGKEICLDPEAPLIKKAIQKILESGNKEN

>Q08782

MQRSSVLLCLLVIEATFCSLLMAQPDGVNTPTCCYTFNKQIPLKRVKGYERITSSRCPQEAVIFRTLKNKEVCADPTQKWVQDYIAKLDQRTQQKQNSTAPQTSKPLNIRFTTQDPKNRS

>Q1RMP9

MEEPQRARSQTVTTTASSFAENFSTTSSSFSYDREFLRTLPGLLIVAEIVLGLLVWTLIAGTEYFRVPAFGWVMFVAVFYWVLTVFFLIIYLTMTYTRIPQVPWTTVGLWFNGSAFALYLSAAIVDASSVSPERDSHNFNSWAASSFFAFLVTICYAGNTYFSFIAWRSRTIQ

>Q29RT9

MMLGRTSRLLLVLLFIAYATTSGNGNEGSKVGSCPCDHTVSSHSPPNENIMRHLRKYLKAYQRCFSYVRFQLPLKNVCGGSTDGWVQELMHCFDSGECGHAQPRVVDAPLHRTQLPEPTEAAPSDTATTSQTYLPSTLQRTQQPTPLEGALSLDSKLIPTHETTTYTSGHSLGAEPEAKENQKQLKENRGPQAGTSATVPVLSLLAIVFILAGVLLYVVCKRRKNQLLQHPPDLAASLYTCSRRTRAENGTL

>Q4PR21

MRPWLLACLVACFVGAWAPAIHAQGAFEDCCLAYHSHIKWRLLRRAHSYQRQDVSGSCNLPAVIFFFPQKDKMVCGKPGAKWVQFGMKILDNRNKKDSKPHHSGRRFFQGPQSGVRKLSSGTSRPLLLKFSGPTRSSKRKASLLTTAIPGP

>Q8IZ96

MDPEHAKPESSEAPSGNLKQPETAAALSLILGALACFIITQANESFITITSLEICIVVFFILIYVLTLHHLLTYLHWPLLDLTNSIITAVFLSVVAILAMQEKKRRHLLYVGGSLCLTAVIVCCIDAFVVTTKMRTNLKRFLGVEVERKLSPAKDAYPETGPDAPQRPA

>Q8TAZ6

MAPKAAKGAKPEPAPAPPPPGAKPEEDKKDGKEPSDKPQKAVQDHKEPSDKPQKAVQPKHEVGTRRGCRRYRWELKDSNKEFWLLGHAEIKIRSLGCLIAAMILLSSLTVHPILRLIITMEISFFSFFILLYSFAIHRYIPFILWPISDLFNDLIACAFLVGAVVFAVRSRRSMNLHYLLAVILIGAAGVFAFIDVCLQRNHFRGKKAKKHMLVPPPGKEKGPQQGKGPEPAKPPEPGKPPGPAKGKK

>Q8UUJ9

MDIRTLALLSILLGTLCLTEGKPVSLVYRCPCRYFESNVPKSNIKHLKILSTSNCSLQIVARLKHNGKQICLDPKTKWIQEYLEKALNKKAKKT

>Q96DZ9

MLSARDRRDRHPEEGVVAELQGFAVDKAFLTSHKGILLETELALTLIIFICFTASISAYMAAALLEFFITLAFLFLYATQYYQRFDRINWPCLLQGHGQSGGPHPLDLLSHSAKVQPQPWPGLTPPGWHTPAAVPWVPAPAPGFWSWLLWFICFHSLGSSDFLRCVSAIIIFLVVSFAAVTSRDGAAIAAFVFGIILVSIFAYDAFKIYRTEMAPGASQGDQQ

>Q96FZ5

MSHGAGLVRTTCSSGSALGPGAGAAQPSASPLEGLLDLSYPRTHAALLKVAQMVTLLIAFICVRSSLWTNYSAYSYFEVVTICDLIMILAFYLVHLFRFYRVLTCISWPLSELLHYLIGTLLLLIASIVAASKSYNQSGLVAGAIFGFMATFLCMASIWLSYKISCVTQSTDAAV

>Q99731

MALLLALSLLVLWTSPAPTLSGTNDAEDCCLSVTQKPIPGYIVRNFHYLLIKDGCRVPAVVFTTLRGRQLCAPPDQPWVERIIQRLQRTSAKMKRRSS

>Q99LJ5

MWPPDAEPEPDPESAHGPRSGRTVPGLRALLPARAFLCSLKGRLLLAESGLSFITFICYVVSSASAFLTVPLLEFLLAVYFLFADAMQLNDKWQGLCWPMMDFLRCVTAALIYFVISITAVAKYSDGAYKAAGVFGFFATIVFAIDFYLIFNEVAKFLKQGDSGNETTAHRTEEENSNSDSDSD

>Q9D6G9

MFSAWDRRERPPEEGAAAGLQGFGVDKTFLSSLKGILLETELALTFIIFICFTASISAYMAAALLEFLITLAFLFLCATQYYQRFDRLNWPCLDFLRCLSAIVIFLVVSFAAVTSREGAAIAAFVFGIILVSVFAYDAFKIYRTELMPSTTEGDQQ

>Q9DAC0

MAAPAPRARTGGKKKDERRGFKGYKWEFRDSNKDFWAQGHAECKSLIMILLIAAMVCFQRVATHPIVILLLTMELSICAFFFFLYSLAINRYIPFVFWPMMDLMNDLACSTFLIGGIFFALEARRELPVPYLTGMILMGVTAFISIIDLCLQRRQFKSRKLRKFILLTPDRKGKKQDPKLLLMLAAKEDEEERQRELAEKAKRESMDPGW

>Q9DAR1

MAAPIKFPFRPRGGQPREDTTPKRGLRRYLLELKESNKEFWLSGHAVFKLLSLGCMISALDYFETMLPHPVLILLICMEAAICIFFIFLNTLAINRYIPFVFWPMADIFNSLFSCVFLGGGIYFAFKARRLLPKPYLTAMILMGAAAICSFIDMLLQFQHFRGLRLRKW

>Q9DAS1

METPRPVVSRRPFCCTLKCFVKFLRLVVTVTSMIFFIVGQAPEPYIVITGFEVTVIFCFLVLYTCGLDKIMRSFFWPLLDVINSMVTALCMLIVSVLALIPETSTKTILGGVFGFLTVTCTIADCALMCQKLRFRPRQPYQKKSTNDIDDRE

>Q9H2A7

MGRDLRPGSRVLLLLLLLLLVYLTQPGNGNEGSVTGSCYCGKRISSDSPPSVQFMNRLRKHLRAYHRCLYYTRFQLLSWSVCGGNKDPWVQELMSCLDLKECGHAYSGIVAHQKHLLPTSPPISQASEGASSDIHTPAQMLLSTLQSTQRPTLPVGSLSSDKELTRPNETTIHTAGHSLAAGPEAGENQKQPEKNAGPTARTSATVPVLCLLAIIFILTAALSYVLCKRRRGQSPQSSPDLPVHYIPVAPDSNT

>Q9JIL2

MQQAGLTLMAVAVCVAFQTSEAILPMASSCCTEVSHHVSGRLLERVSSCSIQRADGDCDLAAVILHVKRRRICISPHNRTLKQWMRASEVKKNGRENVCSGKKQPSRKDRKGHTTRKHRTRGTHRHEASR

>Q9WUQ5

MRLLAAALLLLLLALCASRVDGSKCKCSRKGPKIRYSDVKKLEMKPKYPHCEEKMVIVTTKSMSRYRGQEHCLHPKLQSTKRFIKWYNAWNEKRRVYEE

>Q9WVL7

MAAQGWSMLLLAVLNLGIFVRPCDTQELRCLCIQEHSEFIPLKLIKNIMVIFETIYCNRKEVIAVPKNGSMICLDPDAPWVKATVGPITNRFLPEDLKQKEFPPAMKLLYSVEHEKPLYLSFGRPENKRIFPFPIRETSRHFADLAHNSDRNFLRDSSEVSLTGSDA

>Q9Z1X0

MMEGLSPASSLPLLLLLLSPAPEAALPLPSSTSCCTQLYRQPLPSRLLRRIVHMELQEADGDCHLQAVVLHLARRSVCVHPQNRSLARWLERQGKRLQGTVPSLNLVLQKKMYSHPQQQN

*****Interferon**

>B5B3U4

MIAQNMTIFFWGVCLLTSGWATYSEASVPENLDKSIDELKAYYIKDDHEIHNAHPVFLRVLKDLKVNLEEPEQNLLMSIIMDTYSRIFTRMENDSLDEATKERIAHVQEHLKKLRENYFPGKSAELKTYAETLWAIKEDDPVIQRKALFELKRVYREATLLKNLKNKERRRRQAKNTKNLKS

>C8AW45

MYCRLNMVYLICALLLIVSLQGTVGARLPQSQKDKEQMLKNVREKIESLQKHYHTTGTEWFGKSVLSSHLHQLNSKASCTCQSLLLDSMLNITETIFQDMRGKAENEETKTSLRDVMTEVKMLRHKYSEEQKVWRELQDIHSVEVNNGKIQKGALNSFLILYDLAY

>H2N2P1

MMMMALMKVILCLWLTVTGVSASYVPQEMNKTIQNLLQHYKIPLVERFNGNPVFPKDSMDGNVEMKMIFMHGVLETYEDLIGHMLKQLPTASPPLGSNQDKPASPGTSNDDAAPVKADVRSKLMYILEKIQFLKTHRYQEQEKLLHRLQNLKKIQMDNRTVQSKALWELPQLFEKASSLADNTMRRRRRRQARNRMRLKA

>K9M1U5

MRPSVWAAVAAGLWVLCTVIAAAPRRCLLSHYRSLEPRTLAAAKALRDRYEEEALSWGQRNCSFRPRRDPPRPSSCARLRHVARGIADAQAVLSGLHRSELLPGAGPILELLAAAGRDVAACLELARPGSSRKVPGAQKRRHKPRRADSPRCRKASVVFNLLRLLTWELRLAAHSGPCL

>P56830

CYLSEDHMLGARENLRLLARMNRLSPHPCLQDRKDFGLPQEMVEGNQLQKDQAISVLHEMLQQCFNLFYTEHSSAAWNTTLLEQLCTGLQQQLEDLDACLGQVMEEKDSDMGRMGPILTVKKYFQGIHVYLKEKEYSDCAWEIIRMEMMRALSSSTTLQKRLRKMGGDLNSL

>Q86WN2

MIIKHFFGTVLVLLASTTIFSLDLKLIIFQQRQVNQESLKLLNKLQTLSIQQCLPHRKNFLLPQKSLSPQQYQKGHTLAILHEMLQQIFSLFRANISLDGWEENHTEKFLIQLHQQLEYLEALMGLEAEKLSGTLGSDNLRLQVKMYFRRIHDYLENQDYSTCAWAIVQVEISRCLFFVFSLTEKLSKQGRPLNDMKQELTTEFRSPR

>Q8IU54

MAAAWTVVLVTLVLGLAVAGPVPTSKPTTTGKGCHIGRFKSLSPQELASFKKARDALEESLKLKNWSCSSPVFPGNWDLRLLQVRERPVALEAELALTLKVLEAAAGPALEDVLDQPLHTLHHILSQLQACIQPQPTAGPRPRGRLHHWLHRLQEAPKKESAGCLEASVTFNLFRLLTRDLKYVADGNLCLRTSTHPEST

>Q9TTB0

QDPYVKEAENLKKYFNAGHSDVADNGTLFLGILKNWKEESDRKIMQSQIVSFYFKLFKNFKDDQSIQKSVETIKEDMNVKFFNSNKKKRDDFEKLTNYSVTDLNVQRKAIHELIQVMAELSPAVKTGKRKRSQMLFRGRRASQ

*****Interleukin**

>A0S0B0

MASKHNADLSSAAMLAALLLCALGAPVEYEPTDSPAGDFSGEEQEVTPDLLSASPVWDLIIGVTAHHQKEFEDEFQQEVKYRFLNHYKLSSLPADCPSANFSKEACLQRLAEGLHTYMVLFKHVEKEYPSSSILLHARYHSGALIGLIKEKMRNPGQVTVPTSRQEQQLLQDMDNPSTFHRKMTAHNILRQLHNFLRNGKVAIRKREMPKQKRRKDDGIIPPIHPSYQMT

>A6N6I9

MTVKSLHVTAMVKYLLLLILGLAFLRETAAQRVPKEGQTFFQKPESCPSVPEGSLKLDLGIINANQRVPLSRNIERRSTSPWNYTVTWDPNRYPSEVVQAQCRHLGCVNAQGKEDIFMNSVPIQQETLVLRRKHQGCSVSFQLEKLLVTVGCTCVKPLIHHVH

>B0ZE70

MRILKPHLRSTSIQCYLCLLLNSHFLTEAGIHVFILGCISAGLPKTEANWQFVIRDLEKIDNIIQSIHIDTTLYTESDAHPSCKVTAMKCFLLELRVISLEFKHHVLNETVENLIFLANDRLSSNGDITETGCKECEELEEKNIKEFLQSFVHIVQMFINTS

>O95760

MKPKMKYSTNKISTAKWKNTASKALCFKLGKSQQKAKEVCPMYFMKLRSGLMIKKEACYFRRETTKRPSLKTGRKHKRHLVLAACQQQSTVECFAFGISGVQKYTRALHDSSITGISPITEYLASLSTYNDQSITFALEDESYEIYVEDLKKDEKKDKVLLSYYESQHPSNESGDGVDGKMLMVTLSPTKDFWLHANNKEHSVELHKCEKPLPDQAFFVLHNMHSNCVSFECKTDPGVFIGVKDNHLALIKVDSSENLCTENILFKLSET

>P01583

MAKVPDMFEDLKNCYSENEEDSSSIDHLSLNQKSFYHVSYGPLHEGCMDQSVSLSISETSKTSKLTFKESMVVVATNGKVLKKRRLSLSQSITDDDLEAIANDSEEEIIKPRSAPFSFLSNVKYNFMRIIKYEFILNDALNQSIIRANDQYLTAAALHNLDEAVKFDMGAYKSSKDDAKITVILRISKTQLYVTAQDEDQPVLLKEMPEIPKTITGSETNLLFFWETHGTKNYFTSVAHPNLFIATKQDYWVCLAGGPPSITDFQILENQA

>P01586

MVLASSTTSIHTMLLLLLMLFHLGLQASISGRDTHRLTRTLNCSSIVKEIIGKLPEPELKTDDEGPSLRNKSFRRVNLSKFVESQGEVDPEDRYVIKSNLQKLNCCLPTSANDSALPGVFIRDLDDFRKKLRFYMVHLNDLETVLTSRPPQPASGSVSPNRGTVEC

>P04351

MYSMQLASCVTLTLVLLVNSAPTSSSTSSSTAEAQQQQQQQQQQQQHLEQLLMDLQELLSRMENYRNLKLPRMLTFKFYLPKQATELKDLQCLEDELGPLRHVLDLTQSKSFQLEDAENFISNIRVTVVKLKGSDNTFECQFDDESATVVDFLRRWIAFCQSIISTSPQ

>P05112

MGLTSQLLPPLFFLLACAGNFVHGHKCDITLQEIIKTLNSLTEQKTLCTELTVTDIFAASKNTTEKETFCRAATVLRQFYSHHEKDTRCLGATAQQFHRHKQLIRFLKRLDRNLWGLAGLNSCPVKEANQSTLENFLERLKTIMREKYSKCSS

>P06740

MSCLPVLLLLQLLVSPGLQAPMTQTTSLKTSWVNCSNMIDEIITHLKQPPLPLLDFNNLNGEDQDILMENNLRRPNLEAFNKAVKSLQNASAIESILKNLPPCLPMATAAPTRHPIRIKDGDWNEFRRKLKFYLKTLENEQAQQMTLSLEIS

>P09920

MAQLSAQRRMKLMALQLLLWQSALWSGREAVPLVTVSALPPSLPLPRSFLLKSLEQVRKIQASGSVLLEQLCATYKLCHPEELVLLGHSLGIPKASLSGCSSQALQQTQCLSQLHSGLCLYQGLLQALSGISPALAPTLDLLQLDVANFATTIWQQMENLGVAPTVQPTQSAMPAFTSAFQRRAGGVLAISYLQGFLETARLALHHLA

>P13232

MFHVSFRYIFGLPPLILVLLPVASSDCDIEGKDGKQYESVLMVSIDQLLDSMKEIGSNCLNNEFNFFKRHICDANKEGMFLFRAARKLRQFLKMNSTGDFDLHLLKVSEGTTILLNCTGQVKGRKPAALGEAQPTKSLEENKSLKEQKKLNDLCFLKRLLQEIKTCWNKILMGTKEH

>P15247

MLVTYILASVLLFSSVLGQRCSTTWGIRDTNYLIENLKDDPPSKCSCSGNVTSCLCLSVPTDDCTTPCYREGLLQLTNATQKSRLLPVFHRVKRIVEVLKNITCPSFSCEKPCNQTMAGNTLSFLKSLLGTFQKTEMQRQKSRP

>P20808

MNCVCRLVLVVLSLWPDTAVAPGPPPGSPRASPDPRAELDSTVLLTRSLLEDTRQLTIQLKDKFPADGDHNLDSLPTLAMSAGALGALQLPSVLTRLRADLLSYLRHVQWLRRAMGSSLKTLEPELGTLQTRLDRLLRRLQLLMSRLALPQLPPDPPAPPLAPPSSTWGGIRAAHAILGGLHLTLDWAVRGLLLLKTRL

>P35225

MHPLLNPLLLALGLMALLLTTVIALTCLGGFASPGPVPPSTALRELIEELVNITQNQKAPLCNGSMVWSINLTAGMYCAALESLINVSGCSAIEKTQRMLSGFCPHKVSAGQFSSLHVRDTKIEVAQFVKDLLLHLKKLFREGRFN

>P35834

MAPLGPTGPLPQSFLLKCLEQMRKVQADGTALQETLCATHQLCHPEELVLLGHALGIPQPPLSSCSSQALQLMGCLRQLHSGLFLYQGLLQALAGISPELAPTLDTLQLDTTDFAINIWQQMEDLGMAPAVPPTQGTMPAFTSAFQRRAGGVLVASNLQSFLELAYRALRHFAKP

>P41693

AENNLKLPKLAEKDKCFQSQFNQETCMTRITTGLQEFQIHLKYLEANYEGNKNNAHSVYISTKHLLQKLRPMNQVEVTTPNPTTDSSLQALFKSQDKWLKHVTIHLILRSLEDFLQFSLRAIRIM

>P48411

MHGSALLCCCLVLLAGVGASRHQSTLLEDDCTHFPASLPHMLRELRAAFGRVKIFFQMKDKLDNILLTGSLLEDFKSYLGCQALSEMIQFYLEEVMPRAENHDPDIKNHVNSLGEKLKTLRLRLRLRRCHRFLPCENKSKAVEQVKSAFSKLQEKGVYKAMSEFDIFINYIETYMTMRMKI

>P83714

MYCLLATPLCLLSLLLPPLSPAAPISPSEPIGQAYSLALYMQKNTSALLQTYLQHQGSPFSDPGFSAPELQLSTLPSAAVSFKTWHAMEDAERLSRAQGAFLALTQHLQLVGDDQSYLNPGSPILLAQLGAARLRAQGLLGNMAAIMTALGLPIPPEEDTLGFVPFGASAFERKCRGYIVTREYGHWTDRAVRDLALLKAKYSA

>Q13007

MNFQQRLQSLWTLARPFCPPLLATASQMQMVVLPCLGFTLLLWSQVSGAQGQEFHFGPCQVKGVVPQKLWEAFWAVKDTMQAQDNITSARLLQQEVLQNVSDAESCYLVHTLLEFYLKTVFKNYHNRTVEVRTLKSFSTLANNFVLIVSQLQPSQENEMFSIRDSAHRRFLLFRRAFKQLDVEAALTKALGEVDILLTWMQKFYKL

>Q60480

FEDLKNCYSENEEYASAIDHLSLNQKSFYDTNYDPLHENRVDEPVSPNPYENSEESNFTLEDSSDSSAVVLTSAHGEVLKKRRLSLNQTMSNEDLEAIANDSEEEIIEPWSVPYSFQSNLKFKYQRSIKKGAVITDAMHQSLIRESNGQHLKAMHVVDRKHEVKFDIDGYVSTATRIRPVTLKISKTQLYVCAQEEGQPVLLKE

>Q61728

MCPSARSLLLLASLVLLEHLGSARNLPRSTPVPAVSQECHNLSQTLLSTVDSALQNAIEILEYYPCSAEEVNHEDITKNRTNTVKACLPQELAQNENCLASRETSFIIKRSSLTSGRTSWNTTLCFSSIYEDLKMYQLELKAISEKLLMDPKGQIYEDKALLAAVDYLMQAVNVNNETVPQTPSPEAPSSNLYRTKTKLCILLHALRIRAVTINRVMSYLNSS

>Q6ZMJ4

MPRGFTWLRYLGIFLGVALGNEPLEMWPLTQNEECTVTGFLRDKLQYRSRLQYMKHYFPINYKISVPYEGVFRIANVTRLQRAQVSERELRYLWVLVSLSATESVQDVLLEGHPSWKYLQEVETLLLNVQQGLTDVEVSPKVESVLSLLNAPGPNLKLVRPKALLDNCFRVMELLYCSCCKQSSVLNWQDCEVPSPQSCSPEPSLQYAATQLYPPPPWSPSSPPHSTGSVRPVRAQGEGLLP

>Q80XG2

VVIFLGTVAHKTSPQRPDRLLIRLRHLVDNVEQLKIYVNDLDPELLPAPQDVKEHCAHSAFACFQKAKLKPANTGSNKTIISDLVTQLRRRLPATKAEKKQQSLVKCPSCDSYEKKTPKEFLE

>Q8NEV9

MGQTAGDLGWRLSLLLLPLLLVQAGVWGFPRPPGRPQLSLQELRREFTVSLHLARKLLSEVRGQAHRFAESHLPGVNLYLLPLGEQLPDVSLTFQAWRRLSDPERLCFISTTLQPFHALLGGLGTQGRWTNMERMQLWAMRLDLRDLQRHLRFQVLAAGFNLPEEEEEEEEEEEEERKGLLPGALGSALQGPAQVSWPQLLSTYRLLHSLELVLSRAVRELLLLSKAGHSVWPLGFPTLSPQP

>Q8QFQ8

MSCEEIAVCAVRLRENLCLYFEELECDAFCKDKTIKRFFRNVNSQLLVVRPDLNVAAFEDVTDQEVKSGSGMYFDIHCYKTTAPSARMPVAFSVQVEDKSYYMCCEKEHGKMVVRFREGEVPKDIPGESNIIFFKKTFTSCSSKAFKFEYSLEQGMFLAFEEEDSLRKLILKKLPREDEVDETTKFVTSHNERHNL

>Q8R460

MFSKHPFSTHISGRETPDFGEVFDLDQQVWIFRNQALVTVPRSHRVTPVSVTILPCKYPESLEQDKGIAIYLGIQNPDKCLFCKEVNGHPTLLLKEEKILDLYHHPEPMKPFLFYHTRTGGTSTFESVAFPGHYIASSKTGNPIFLTSKKGEYYNINFNLDIKS

>Q8TAD2

MLVAGFLLALPPSWAAGAPRAGRRPARPRGCADRPEELLEQLYGRLAAGVLSAFHHTLQLGPREQARNASCPAGGRPADRRFRPPTNLRSVSPWAYRISYDPARYPRYLPEAYCLCRGCLTGLFGEEDVRFRSAPVYMPTVVLRRTPACAGGRSVYTEAYVTIPVGCTCVPEPEKDADSINSSIDKQGAKLLLGPNDAPAGP

>Q90YI0

MNFTEGCEATGRRPGSAGSRRRRAPRPGPVALLPLLLPLLLPPAAAVPLPAAADSSGEVGLEEEAGARRALLDCEPLARVLRDRAVQLQDEMCKKFTVCENSMEMLVRNNLNLPKVTEEDGCLLAGFDEEKCLTKLSSGLFAFQTYLEFIQETFDSEKQNVESLCYSTKHLAATIRQMVINPDEVVIPDSAAQKSLLANLKSDKDWIEKITMHLILRDFTSFMEKTVRAVRYLKKTRSFSA

>Q91Z84

MLDCRAIILLWLLPWATQGLAVPRSSSPDWAQCQQLSRNLCTLAWSAHTPVGQMDLLREEGEEETKSDVPRIQCGDGCDPQGLKDNSQFCLQRIRQGLVFYKHLLDSDIFTGEPSLLPDSPVDQLHTSLLGLSQLLQPEDHHWETQQMPRLSPSQQWQRSLLRSKILRSLQAFLAIAARVFAHGAATLTEPLVPTA

>Q9D6Z6

MMAFPPQSCVHVLPPKSIQMWEPNHNTMHGSSQSPRNYRVHDSQQMVWVLTGNTLTAVPASNNVKPVILSLIACRDTEFQDVKKGNLVFLGIKNRNLCFCCVEMEGKPTLQLKEVDIMNLYKERKAQKAFLFYHGIEGSTSVFQSVLYPGWFIATSSIERQTIILTHQRGKLVNTNFYIESEK

>Q9GZX6

MAALQKSVSSFLMGTLATSCLLLLALLVQGGAAAPISSHCRLDKSNFQQPYITNRTFMLAKEASLADNNTDVRLIGEKLFHGVSMSERCYLMKQVLNFTLEEVLFPQSDRFQPYMQEVVPFLARLSNRLSTCHIEGDDLHIQRNVQKLKDTVKKLGESGEIKAIGELDLLFMSLRNACI

>Q9H293

MRERPRLGEDSSLISLFLQVVAFLAMVMGTHTYSHWPSCCPSKGQDTSEELLRWSTVPVPPLEPARPNRHPESCRASEDGPLNSRAISPWRYELDRDLNRLPQDLYHARCLCPHCVSLQTGSHMDPRGNSELLYHNQTVFYRRPCHGEKGTHKGYCLERRLYRVSLACVCVRPRVMG

>Q9HBE4

MRSSPGNMERIVICLMVIFLGTLVHKSSSQGQDRHMIRMRQLIDIVDQLKNYVNDLVPEFLPAPEDVETNCEWSAFSCFQKAQLKSANTGNNERIINVSIKKLKRKPPSTNAGRRQKHRLTCPSCDSYEKKPPKEFLERFKSLLQKMIHQHLSSRTHGSEDS

>Q9JI24

MQTSLRQQILPGLSLILLVLNQVPELQGQEFRFGPCQVTGVVLPELWEAFWTVKNTVKTQDELTSVRLLKPQVLQNVSDAESCYLAHSLLKFYLNTVFKNYHSKIVKFKVLKSFSTLANNFLVIMSKLQPSKDNAMLPISDSARRRFLLYHRTFKQLDIEVALAKAFGEVDILLAWMQNFYQL

>Q9JLA2

MNKEKELRAASPSLRHVQDLSSRVWILQNNILTAVPRKEQTVPVTITLLPCQYLDTLETNRGDPTYMGVQRPMSCLFCTKDGEQPVLQLGEGNIMEMYNKKEPVKASLFYHKKSGTTSTFESAAFPGWFIAVCSKGSCPLILTQELGEIFITDFEMIVVH

>Q9MZR1

MNSFTSALRPGPLGCSLALLLVVATAFPTSAPVREDSNTKASPDKTLTPPGRTIESIRSILETIKELRKEMCDHDVNCMNRKEALAEVNLHLPRLIEEDGCFPPAVNNETCLLRITSGLMEFRMYLEHLQAKFRSDEENTRVSMVLKNIQHLIKTLRPKVKNLNEEATLKPAVAVSLMENLQQKNQWLKTTTIHFILRGLTNFLEFTLRAVDLMECGCPCLRNFMGSASHGQNTPSCPLDT

>Q9NPH9

MLVNFILRCGLLLVTLSLAIAKHKQSSFTKSCYPRGTLSQAVDALYIKAAWLKATIPEDRIKNIRLLKKKTKKQFMKNCQFQEQLLSFFMEDVFGQLQLQGCKKIRFVEDFHSLRQKLSHCISCASSAREMKSITRMKRIFYRIGNKGIYKAISELDILLSWIKKLLESSQ

>Q9NZH6

MSFVGENSGVKMGSEDWEKDEPQCCLEDPAGSPLEPGPSLPTMNFVHTSPKVKNLNPKKFSIHDQDHKVLVLDSGNLIAVPDKNYIRPEIFFALASSLSSASAEKGSPILLGVSKGEFCLYCDKDKGQSHPSLQLKKEKLMKLAAQKESARRPFIFYRAQVGSWNMLESAAHPGWFICTSCNCNEPVGVTDKFENRKHIEFSFQPVCKAEMSPSEVSD

>Q9NZH7

MNPQREAAPKSYAIRDSRQMVWVLSGNSLIAAPLSRSIKPVTLHLIACRDTEFSDKEKGNMVYLGIKGKDLCLFCAEIQGKPTLQLKLQGSQDNIGKDTCWKLVGIHTCINLDVRESCFMGTLDQWGIGVGRKKWKSSFQHHHLRKKDKDFSSMRTNIGMPGRM

>Q9NZH8

MRGTPGDADGGGRAVYQSMCKPITGTINDLNQQVWTLQGQNLVAVPRSDSVTPVTVAVITCKYPEALEQGRGDPIYLGIQNPEMCLYCEKVGEQPTLQLKEQKIMDLYGQPEPVKPFLFYRAKTGRTSTLESVAFPDWFIASSKRDQPIILTSELGKSYNTAFELNIND

>Q9P0M4

MTLLPGLLFLTWLHTCLAHHDPSLRGHPHSHGTPHCYSAEELPLGQAPPHLLARGAKWGQALPVALVSSLEAASHRGRHERPSATTQCPVLRPEEVLEADTHQRSISPWRYRVDTDEDRYPQKLAFAECLCRGCIDARTGRETAALNSVRLLQSLLVLRRRPCSRDGSGLPTPGAFAFHTEFIHVPVGCTCVLPRSV

>Q9QXT6

MDWPHSLLFLLAISIFLAPSHPRNTKGKRKGQGRPSPLAPGPHQVPLDLVSRVKPYARMEEYERNLGEMVAQLRNSSEPAKKKCEVNLQLWLSNKRSLSPWGYSINHDPSRIPADLPEARCLCLGCVNPFTMQEDRSMVSVPVFSQVPVRRRLCPQPPRPGPCRQRVVMETIAVGCTCIF

>Q9QYY1

MMVLSGALCFRMKDSALKVLYLHNNQLLAGGLHAEKVIKGEEISVVPNRALDASLSPVILGVQGGSQCLSCGTEKGPILKLEPVNIMELYLGAKESKSFTFYRRDMGLTSSFESAAYPGWFLCTSPEADQPVRLTQIPEDPAWDAPITDFYFQQCD

>Q9QZM3

MDLRAGDSWGMLACLCTVLWHLPAVPALNRTGDPGPGPSIQKTYDLTRYLEHQLRSLAGTYLNYLGPPFNEPDFNPPRLGAETLPRATVNLEVWRSLNDRLRLTQNYEAYSHLLCYLRGLNRQAATAELRRSLAHFCTSLQGLLGSIAGVMATLGYPLPQPLPGTEPAWAPGPAHSDFLQKMDDFWLLKELQTWLWRSAKDFNRLKKKMQPPAASVTLHLEAHGF

>Q9XT91

MMKILVCLPLLTLYAGCVYGIATGNPVSRLVTETLSLITAHRTLLIGNGTLRISIPDPQNHPLCIEEIFQGIETLKNQTAEENVVEKIFQNLSSLKGYITAKEKQCGGERRRVEQFLDYLEEFLRTINIEWNTEWTVES

>Q9YGD3

MDFESNYSLIKNTSESAAWSSKLPQGLDLEVSHHPITMRHIANLIIAMERLKGGEGVTMGTEFKDKDLLNFLLESAVEEHIVLELESAPPASRRAAGFSSTSQYECSVTDSENKCWVLMNEAMELHAMMLQGGSSYHKVHLNLSSYVTPVPIETEARPVALGIKGSNLYLSCSKSGGRPTLHLEEVADKDQLKSISQQSDMVRFLFYRRNTGVDISTLESASFRNWFISTDMQQDYTKPVDMCQKAAPNRLTTFTIQRHN

*****Others**

>A9JSE7

MPTFTVCTNVCRDSMPDTLLSDLTKLLAKATGKPAEYIAIHIMPDQMMSFGDSTDPCAVCSLSSIGKIGGPQNKSYSKLLCDYLTKQMNIPANRVYINFHDLNPANVGWNGSTFA

>M0R7X9

MAPSPRTSSRQDATALPSMSSTFWAFMILASLLIAYCSQLAAGTCEIVTLDRDSSQPRRTIARQTARCACRKGQIAGTTRARPACVDARIIKTKQWCDMLPCLEGEGCDLLINRSGWTCTQPGGRIKTTTVS

>O35793

MNRTAYTVGALLLLLGTLLPAAEGKKKGSQGAIPPPDKAQHNDSEQTQSPPQPGSRTRGRGQGRGTAMPGEEVLESSQEALHVTERKYLKRDWCKTQPLKQTIHEEGCNSRTIINRFCYGQCNSFYIPRHIRKEEGSFQSCSFCKPKKFTTMMVTLNCPELQPPTKKKRVTRVKQCRCISIDLD

>O55233

MHLLLVQLLVLLPLGKADLCVDGCQSQGSLSFPLLERGRRDLHVANHEEAEDKPDLFVAVPHLMGTSLAGEGQRQRGKMLSRLGRFWKKPETEFYPPRDVESDHVSSGMQAVTQPADGRKVERSPLQEEAKRFWHRFMFRKGPAFQGVILPIKSHEVHWETCRTVPFNQTIAHEDCQKVVVQNNLCFGKCSSIRFPGEGADAHSFCSHCSPTKFTTVHLMLNCTSPTPVVKMVMQVEECQCMVKTERGEERLLLAGSQGSFIPGLPASKTNP

>O62757

MWLQNLLFLNTVVCSISAPTSSPSSVTRPWQHVDAMKEALSLLNNSSEITAVMNETVEVVSEMFDPEEPKCLQTHLKLYEQGLRGSLISLKEPLRMMANHYKQHCPLTPETPCETQTITFKNFKEKLKDFLFNNPFDCWGPDQK

>O88593

MLFACALLALLGLATSCSFIVPRSEWRALPSECSSRLGHPVRYVVISHTAGSFCNSPDSCEQQARNVQHYHKNELGWCDVAYNFLIGEDGHVYEGRGWNIKGDHTGPIWNPMSIGITFMGNFMDRVPAKRALRAALNLLECGVSRGFLRSNYEVKGHRDVQSTLSPGDQLYQVIQSWEHYRE

>P06744

MAALTRDPQFQKLQQWYREHRSELNLRRLFDANKDRFNHFSLTLNTNHGHILVDYSKNLVTEDVMRMLVDLAKSRGVEAARERMFNGEKINYTEGRAVLHVALRNRSNTPILVDGKDVMPEVNKVLDKMKSFCQRVRSGDWKGYTGKTITDVINIGIGGSDLGPLMVTEALKPYSSGGPRVWYVSNIDGTHIAKTLAQLNPESSLFIIASKTFTTQETITNAETAKEWFLQAAKDPSAVAKHFVALSTNTTKVKEFGIDPQNMFEFWDWVGGRYSLWSAIGLSIALHVGFDNFEQLLSGAHWMDQHFRTTPLEKNAPVLLALLGIWYINCFGCETHAMLPYDQYLHRFAAYFQQGDMESNGKYITKSGTRVDHQTGPIVWGEPGTNGQHAFYQLIHQGTKMIPCDFLIPVQTQHPIRKGLHHKILLANFLAQTEALMRGKSTEEARKELQAAGKSPEDLERLLPHKVFEGNRPTNSIVFTKLTPFMLGALVAMYEHKIFVQGIIWDINSFDQWGVELGKQLAKKIEPELDGSAQVTSHDASTNGLINFIKQQREARVQ

>P08721

MRLAVVCFCLFGLASCLPVKVAEFGSSEEKAHYSKHSDAVATWLKPDPSQKQNLLAPQNSVSSEETDDFKQETLPSNSNESHDHMDDDDDDDDDGDHAESEDSVNSDESDESHHSDESDESFTASTQADVLTPIAPTVDVPDGRGDSLAYGLRSKSRSFPVSDEQYPDATDEDLTSRMKSQESDEAIKVIPVAQRLSVPSDQDSNGKTSHESSQLDEPSVETHSLEQSKEYKQRASHESTEQSDAIDSAEKPDAIDSAERSDAIDSQASSKASLEHQSHEFHSHEDKLVLDPKSKEDDRYLKFRISHELESSSSEVN

>P09056

MKVLAAGIVPLLLLVLHWKHGAGSPLPITPVNATCAIRHPCHGNLMNQIKNQLAQLNGSANALFISYYTAQGEPFPNNVEKLCAPNMTDFPSFHGNGTEKTKLVELYRMVAYLSASLTNITRDQKVLNPTAVSLQVKLNATIDVMRGLLSNVLCRLCNKYRVGHVDVPPVPDHSDKEAFQRKKLGCQLLGTYKQVISVVVQAF

>P15514

MRAPLLPPAPVVLSLLILGSGHYAAGLDLNDTYSGKREPFSGDHSADGFEVTSRSEMSSGSEISPVSEMPSSSEPSSGADYDYSEEYDNEPQIPGYIVDDSVRVEQVVKPPQNKTESENTSDKPKRKKKGGKNGKNRRNRKKKNPCNAEFQNFCIHGECKYIEHLEAVTCKCQQEYFGERCGEKSMKTHSMIDSSLSKIALAAIAAFMSAVILTAVAVITVQLRRQYVRKYEGEAEERKKLRQENGNVHAIA

>P22800

ENFSGGCVAGYMRTPDGRCKPTFYQ

>P28797

MWTLVGWTILVAGLVAGIRCPDDQVCPVACCPDSGGASYSCCDPGVDLRTTALSGYLGRPCQSPANCPIGHSCVLTAAGTAACCPFSQAMACGDGHHCCPYGFHCSTDGGTCIQRPDIHLLGAVQCPGGEFECPDSSTCCHMLDGSWGCCPMPQASCCEDRVHCCPHGASCDLVHIRCVTALGSHPLTTKLPAQRTNYTGAEGTPVVSPGLLPAALPTSVICPDSRSQCPDDTTCCLLASGEYGCCPMPNAICCSDHLHCCPQDTVCDLRQSRCLSQNKAKTLLTKLPSWTVWDVECDQEVSCPEGQTCCRLQSGKWGCCPFPKAVCCEDHVHCCPEGFRCHTEKDTCEQGLLQVPWAQKTPAQPSRPSQPSPPGPPGPPSPPGPLRSEISCDEVVSCAPGNICCRLASGEWGCCPSSEGYLCMAGERCQVGDRLAPEKMAAHLMSLSQTTDVGCDQHASCPVGQTCCPKLGGGWACCQLPHAVCCEDGQHCCPAGYTCNVKARSCEKAADGAHLAAPLAVGSTGGVMDVACGDRHFCHDEQTCCRDSRGGWACCPFHQGVCCKDQRHCCPAGFHCESQGTRCVHKKSLLHWDSLPRPAAPRPRL

>P30251

ENFSGGCIPGYMRTADGRCKPTY

>P30253

ENFAGGCATGYLRTADGRCKPTF

>P30255

ENFAGGCTPGYQRTADGRCKATF

>P40226

MELTDLLLAAMLLAVARLTLSSPVAPACDPRLLNKLLRDSHLLHSRLSQCPDVDPLSIPVLLPAVDFSLGEWKTQTEQSKAQDILGAVSLLLEGVMAARGQLEPSCLSSLLGQLSGQVRLLLGALQGLLGTQLPLQGRTTAHKDPNALFLSLQQLLRGKVRFLLLVEGPTLCVRRTLPTTAVPSSTSQLLTLNKFPNRTSGLLETNFSVTARTAGPGLLSRLQGFRVKITPGQLNQTSRSPVQISGYLNRTHGPVNGTHGLFAGTSLQTLEASDISPGAFNKGSLAFNLQGGLPPSPSLAPDGHTPFPPSPALPTTHGSPPQLHPLFPDPSTTMPNSTAPHPVTMYPHPRNLSQET

>P42706

SPAPPACDPRLLNKLLRDDHVLHGRL

>P43490

MNPAAEAEFNILLATDSYKVTHYKQYPPNTSKVYSYFECREKKTENSKLRKVKYEETVFYGLQYILNKYLKGKVVTKEKIQEAKDVYKEHFQDDVFNEKGWNYILEKYDGHLPIEIKAVPEGFVIPRGNVLFTVENTDPECYWLTNWIETILVQSWYPITVATNSREQKKILAKYLLETSGNLDGLEYKLHDFGYRGVSSQETAGIGASAHLVNFKGTDTVAGLALIKKYYGTKDPVPGYSVPAAEHSTITAWGKDHEKDAFEHIVTQFSSVPVSVVSDSYDIYNACEKIWGEDLRHLIVSRSTQAPLIIRPDSGNPLDTVLKVLEILGKKFPVTENSKGYKLLPPYLRVIQGDGVDINTLQEIVEGMKQKMWSIENIAFGSGGGLLQKLTRDLLNCSFKCSYVVTNGLGINVFKDPVADPNKRSKKGRLSLHRTPAGNFVTLEEGKGDLEEYGQDLLHTVFKNGKVTKSYSFDEIRKNAQLNIELEAAHH

>P53347

MQTRLLRTLLSLTLSLLILSMALANRGCSNSSSQLLSQLQNQANLTGNTESLLEPYIRLQNLNTPDLRAACTQHSVAFPSEDTLRQLSKPHFLSTVYTTLDRVLYQLDALRQKFLKTPAFPKLDSARHNILGIRNNVFCMARLLNHSLEIPEPTQTDSGASRSTTTPDVFNTKIGSCGFLWGYHRFMGSVGRVFREWDDGSTRSRRQSPLRARRKGTRRIRVRHKGTRRIRVRRKGTRRIWVRRKGSRKIRPSRSTQSPTTRA

>P58499

MRPLAGGLLKVVFVVFASLCAWYSGYLLAELIPDAPLSSAAYSIRSIGERPVLKAPVPKRQKCDHWTPCPSDTYAYRLLSGGGRSKYAKICFEDNLLMGEQLGNVARGINIAIVNYVTGNVTATRCFDMYEGDNSGPMTKFIQSAAPKSLLFMVTYDDGSTRLNNDAKNAIEALGSKEIRNMKFRSSWVFIAAKGLELPSEIQREKINHSDAKNNRYSGWPAEIQIEGCIPKERS

>P81013

VIHCDAATICPDGTTCCLSPYGVWYCCPFSMGQCCRDGIHCCRHGYHCDSTSTHCLR

>P81530

PIFTFASNVPADTITGFFL

>Q27913

MKLTISILFCVILTLQYNGADGKLKDLFGKIHDSVHGTADKVKEDLNSLFHPNDKNQQGNNDASSNIHFADSEENTDAAKKPDEVTPATTTTTTAAPAVPNAPSDNPTTLAPSTTTKDGRENFSGGCVAGYMRTPDGRCKPTFYQ

>Q6DF53

MTVWLLIGFLLPVAIFAAPPINRLALFPDKSAWCEAKNITQIVGHSGCESKSIQNRACLGQCFSYSVPNTFPQSTESLVHCDSCMPIDSVWDVVTLECPGNEEFPRVDKLVEKILQCSCQACGKELSQEGAMFNVYLNTAEETLSPAETLGHHHHRPPAREEDSPAQSQREGESEE

>Q920D7

MKLTTTFLVLCVALLSDSGVAFFMDSLAKPAVEPVAALAPAAEAVAGAVPSLPLSHLAILRFILASMGIPLDPLIEGSRKCVTELGPEAVGAVKSLLGVLTMFG

>Q9VAK8

MASPVVSLLLVGICALAFVHVARSECCTSRELVEFKMDRGDCEAVRAIENYPNGCEVTICADGVAQLGAYCGQGSCNIFGCNCDGGCLSGDWSQEFVRRNQQYGIQIIKVTRLPF

*****TGF-beta**

>O60542

MAVGKFLLGSLLLLSLQLGQGWGPDARGVPVADGEFSSEQVAKAGGTWLGTHRPLARLRRALSGPCQLWSLTLSVAELGLGYASEEKVIFRYCAGSCPRGARTQHGLALARLQGQGRAHGGPCCRPTRYTDVAFLDDRHRWQRLPQLSAAACGCGG

>O61643

MRFAFDSNHSQSGAPFKGSRCFFNCQCICCRQGCCVVVVKCCCCFNLNCCNSLGSRKSFPQPAAMRKKVADLEVLRVSRFVAVILVLARWVTAVATLLTSCILLDIFSVPGQSGVADRSQASSRTVHVSVPTTPNETPSSTSETKLKLLYGYTSYDINNDQQVKSNNLCRVLCKSRNRKRQRRRRRRRNHRRRRHRYTKRLHHLMQDNMSGFEQRLNFSDAKCQSLETNYGTNYDLVQGGKLFSQSERSLLVSPLREIEAPWPAIHGSMRNCSKIKRNRANLIWLLIGLVWFEVKLINCNGISSSNYYASNLESHKGCTLCHESGKPNIYTDKDNPHTDYNIYNKYHSNNNFNKKTNQPHNNIAPSDEVRLESIKRQILTKLGLSHKPNVSHPLPKQFIWETIYRVDGGRMIPNNAFGSSGKNLDQKTIKLRAFASPGSHLFNGRGGRTDQRSERDPSHHKYRSPFDFTFNISKNNVYGKVLRNRSLERIDKKNSFLNGWTENRQLKINSQIASMPIELKSHHNSSPKELKSGAVRKVNGINGTQMNENALKKSTYPIDINHSIDNKTHTGKNGEMSHNDYEYFNDYSVQTHDKNRYHEGRSSIGYQPAIHNIEYENQKGHHESFADDHENIDHEDFFGNTQEIITFAEEGTQYRQYRILEFSAQNRRVPSQKLSIRSAQIHIRIDKPHSLWIEKAKSLPEKHLLNTKRKWGANKPHHRIKIWVFQLSTSINITEKGIDKAIIFRASFQVDPKNLGWQKFDLTDTIREWYGHTSHEKLRLLIDCTGCGGRYSLHLFQTSKLRGNSSDYLSTNPNRPFLVLHTESSRTRRVRRRAVDCGGALNGQCCKESFYVSFKALGWDDWIIAPRGYFANYCRGDCTGSFRTPDTFQTFHAHFIEEYRKMGLMNGMRPCCAPIKFSSMSLIYYGDDGIIKRDLPKMVVDECGCP

>P03972

MPGPSLSLALVLSAMGALLRPGTPREEVFSTSALPREQATGSGALIFQQAWDWPLSSLWLPGSPLDPLCLVTLHGSGNGSRAPLRVVGVLSSYEQAFLEAVRRTHWGLSDLTTFAVCPAGNGQPVLPHLQRLQAWLGEPGGRWLVVLHLEEVTWEPTPLLRFQEPPPGGASPPELALLVVYPGPGLEVTVTGAGLPGTQSLCLTADSDFLALVVDHPEGAWRRPGLALTLRRRGNGALLSTAQLQALLFGADSRCFTRKTPALLLLLPARSSAPMPAHGRLDLVPFPQPRASPEPEEAPPSADPFLETLTRLVRALAGPPARASPPRLALDPGALAGFPQGQVNLSDPAALERLLDGEEPLLLLLPPTAATTGVPATPQGPKSPLWAAGLARRVAAELQAVAAELRALPGLPPAAPPLLARLLALCPGNPDSPGGPLRALLLLKALQGLRAEWRGRERSGSARAQRSAGAAAADGPCALRELSVDLRAERSVLIPETYQANNCQGACGWPQSDRNPRYGNHVVLLLKMQARGATLARPPCCVPTAYTGKLLISLSEERISAHHVPNMVATECGCR

>P22004

MPGLGRRAQWLCWWWGLLCSCCGPPPLRPPLPAAAAAAAGGQLLGDGGSPGRTEQPPPSPQSSSGFLYRRLKTQEKREMQKEILSVLGLPHRPRPLHGLQQPQPPALRQQEEQQQQQQLPRGEPPPGRLKSAPLFMLDLYNALSADNDEDGASEGERQQSWPHEAASSSQRRQPPPGAAHPLNRKSLLAPGSGSGGASPLTSAQDSAFLNDADMVMSFVNLVEYDKEFSPRQRHHKEFKFNLSQIPEGEVVTAAEFRIYKDCVMGSFKNQTFLISIYQVLQEHQHRDSDLFLLDTRVVWASEEGWLEFDITATSNLWVVTPQHNMGLQLSVVTRDGVHVHPRAAGLVGRDGPYDKQPFMVAFFKVSEVHVRTTRSASSRRRQQSRNRSTQSQDVARVSSASDYNSSELKTACRKHELYVSFQDLGWQDWIIAPKGYAANYCDGECSFPLNAHMNATNHAIVQTLVHLMNPEYVPKPCCAPTKLNAISVLYFDDNSNVILKKYRNMVVRACGCH

>P34819

GKHNSAPMFMLDLYNAMAVEEGGGPAGQGFSYPYKAVFSTQGPPLASLQDSHFLTDADMVMSFVNLVEHDKEFFHPRYHHREFRFDLSKIPEGEAVTAAEFRIYKDYIRERFDNETFRISVYQVLQEHLGRESDLFLLDSRTLWASEEGWLVFDITATSNHWVVNPRHNLGLQLCVETLDGQSINPK

>P38440

DAGEAEEGLFTYVFQPSQHTRSRQVTSAQLWFHTGLDRQETAAANSSEPLLGLLVLTSGGPMPVPMSLGQAPPRWAVLHLATSAFPLLTHPVLALLLRCPLCSCSARPEATPFLVAHTRAKPPSGGERARRSTPPLPWPWSPAALRLLQRPPEEPAAHADCHRAALNISFQELGWDRWIVHPPSFIFYYCHGGCGLPTLQDLPLPVPGVPPTPFQPLSLVPGAQACCAALPGTMRPLRVRTTSDGGYSFKYEMVPNLLTQHCACI

>P43021

MSAHSLRILLLQACWALLHPRAPTAAALPLWTRGQPSSPSPLAYMLSLYRDPLPRADIIRSLQAQDVDVTGQNWTFTFDFSFLSQEEDLVWAELRLQLPGPMDIPTEGPLTIDIFHQAKGDPERDPADCLERIWMETFTVIPSQVTFASGSTVLEVTKPLSKWLKDPRALEKQVSSRAEKCWHQPYTPPVPVASTNVLMLYSNRPQEQRQLGGATLLWEAESSWRAQEGQLSVERGGWGRRQRRHHLPDRSQLCRRVKFQVDFNLIGWGSWIIYPKQYNAYRCEGECPNPVGEEFHPTNHAYIQSLLKRYQPHRVPSTCCAPVKTKPLSMLYVDNGRVLLEHHKDMIVEECGCL

>P43026

MRLPKLLTFLLWYLAWLDLEFICTVLGAPDLGQRPQGTRPGLAKAEAKERPPLARNVFRPGGHSYGGGATNANARAKGGTGQTGGLTQPKKDEPKKLPPRPGGPEPKPGHPPQTRQATARTVTPKGQLPGGKAPPKAGSVPSSFLLKKAREPGPPREPKEPFRPPPITPHEYMLSLYRTLSDADRKGGNSSVKLEAGLANTITSFIDKGQDDRGPVVRKQRYVFDISALEKDGLLGAELRILRKKPSDTAKPAAPGGGRAAQLKLSSCPSGRQPASLLDVRSVPGLDGSGWEVFDIWKLFRNFKNSAQLCLELEAWERGRAVDLRGLGFDRAARQVHEKALFLVFGRTKKRDLFFNEIKARSGQDDKTVYEYLFSQRRKRRAPLATRQGKRPSKNLKARCSRKALHVNFKDMGWDDWIIAPLEYEAFHCEGLCEFPLRSHLEPTNHAVIQTLMNSMDPESTPPTCCVPTRLSPISILFIDSANNVVYKQYEDMVVESCGCR

>P48969

MEYSRKTYLDLNIMAKYILILSLFFGPGLSWDVFYSGDEDQLSLARERRAANYNPSPHMSTWERNEIQQEILNILGLQHRPRPPSLRGGQNQFCAQFTEWSYYRTLNIDEQSGHPSETEPQPGGLASNAIYNSPDSSGIGSVMSGTVFNYTRNEVQAVSQADTIMSLPVHYKDAAIEDTEHRYRFDIGRIPQGETVTSAELRVFRDAGRQGRSLYRIDVLLLRERGSDGSRSPVYLDSTIVGAGDHGWLVFDMTSATSTWRSYPGANVGLQLRVESLQGLNIDPTDAGVVGVGNNEGREPFMVVFFQRNEEVIATNSHLRRNRRAATRQKKGGKRPRKPDTDNDIASRDSASSLNSDWQCKRKNLFVNFEDLDWQEWIIAPLGYVAFYCQGECAFPLNGHANATNHAIVQTLVHHMSPSHVPQPCCAPTKLSPITVLYYDDSRNVVLKKYKNMVVRACGCL

>P55101

MVPPLPLLLLLLLVPQGGHGCQGSELDREIVLAKVRALFLDALGPPAVTGEGGDPGVRRLPRRHALGGFARRGSEPEEEDVSQAILFPASGSRCEDEPAAGELAQEAEQGLFTYMFRPSQHMRSRQVTSAHLWFHTGLDRQGTAASNSSEPLLGLLALSSGGPMAVPVTLGQAPPCWAVLHLAASALPLLTHPVLVLLLRCPLCSCSARPEATPFLVAHTRARPPSGGERTRRSTPPLPWPWSPAALRLLQRPPEEPAAHANCHRAALNISFQELGWDRWIVHPRSFIFHYCHGGCGLSAPPDLPLPVPEVPPTPIQPLSLVPGAQPCCAALPGTMRPLRVRTTSDGGYSFKYEIVPNLLTQHCACI

>P55106

MDTSRVLLSAVFLISFLWDLPGFQQASISSSSSSAELGSAKGMRSRKEGRMPRAPRENATAREPLDRQEPPPRPQEEPQRRPPQQPEAREPPGRGPRVVPHEYMLSIYRTYSIAEKLGINASFFQSSKSANTITSFVDRGLDDLSHTPLRRQKYLFDVSTLSDKEELVGAELRLFRQAPAAPWGPPAGPLRLQLFACQSPLLLEARSLDPQGAPRPGWEVFDVWRGLRPQPWKQLCLELRAAWGGEPGAAEDEARAPGPQQPPPPDLRSLGFGRRVRTPQERALLVVFSRSQRKTLFAEMREQLGSATEVVGPGGGAEGSGPPPPPPPPPPSGTPDAGLWSPSPGRRRRRTAFASRHGKRHGKKSRLRCSKKPLHVNFKELGWDDWIIAPLEYEAYHCEGVCDFPLRSHLEPTNHAIIQTLMNSMDPGSTPPSCCVPTKLTPISILYIDAGNNVVYKQYEEMVVESCGCR

>P55107

MAHVPARTSPGPGPQLLLLLLPLFLLLLRDVAGSHRAPAWSALPAAADGLQGDRDLQRHPGDAAATLGPSAQDMVAVHMHRLYEKYSRQGARPGGGNTVRSFRARLEVVDQKAVYFFNLTSMQDSEMILTATFHFYSEPPRWPRALEVLCKPRAKNASGRPLPLGPPTRQHLLFRSLSQNTATQGLLRGAMALAPPPRGLWQAKDISPIVKAARRDGELLLSAQLDSEERDPGVPRPSPYAPYILVYANDLAISEPNSVAVTLQRYDPFPAGDPEPRAAPNNSADPRVRRAAQATGPLQDNELPGLDERPPRAHAQHFHKHQLWPSPFRALKPRPGRKDRRKKGQEVFMAASQVLDFDEKTMQKARRKQWDEPRVCSRRYLKVDFADIGWNEWIISPKSFDAYYCAGACEFPMPKIVRPSNHATIQSIVRAVGIIPGIPEPCCVPDKMNSLGVLFLDENRNVVLKVYPNMSVDTCACR

>P91699

MRAWILLLAVLATSQPIVQVASTEDTSISQRFIAAIAPTRTEPSAASAAAAAATATATATATTALAKAFNPFNELLYKSSDSDSDNNNNNYKNRNNNNNNLNKGPRNNKNKGNKHSKSDANRQFNEVHKPRTDQLENSKNKPKQLVNKTNKMAVKDQKHHQPQQQQQQHHKPATTTALTSTESHQSPIETIFVDDPALALEEEVASINVPANAGAIIEEQEPSTYSKKELIKDKLKPDPSTLVEIENSLLSLFNMKRPPKIDRSKIIIPEAMKKLYAEIMGHELDSVNIPRPGLLTKSANTVRSFTHKDSKIDDRFPHHHRFRLHFDVKSIPAEEKLKAAELQLTRDALAQAAVASTSANRTRYQVLVYDITRVGVRGQREPSYLLLDTKTVRLNSTDTVSLDVQPAVDRWLATPQKNYGLLVEVRTMRSLKPAPHHHVRLRRSADEAHEQWQHKQPLLFAYTDDGRHKARSIRDVSGGGGGGGGAGEGGKGNGGGRNRRHQRRPARRKNHEETCRRHSLYVDFADVGWDDWIVAPPGYDAYYCHGKCPFPLADHFNSTNHAVVQTLVNNLNPGKVPKACCVPTQLDSVAMLYLNDQSTVVLKNYQEMTVVGCGCR

>Q6AYE8

MELGLGEPTALSHCLRPRWQPALWPTLAALALLSSVTEASLDPMSRSPASRDVPSPVLAPPTDYLPGGHTAHLCSERALRPPPQSPQPAPPPPGPALQSPPAALRGARAARAGTRSSRARATDARGCRLRSQLVPVSALGLGHSSDELIRFRFCSGSCRRARSPHDLSLASLLDAGALRSPPGSRPISQPCCRPTRYEAVSFMDVNSTWRTVDHLSATACGCLG

>Q90XB8

MHLYFSCFILLFVPGGKSLGINSHLKHMSNKSQDQVNRTRTVGSKDVAALPLSSYMFNLYQSFHHSELNHGMEAAPSLSLNHRADIIRSLAVKSYDHGGSLWTFLFDFSSLSQEEEHQFAEVRFDFRAFSDAILVGMEVIVDFFHQSSTCQSISGFCQSYLYVGSLTSTLWPRSSDTWVTFEATDIIHKWFERNDKGKNHSEGHMKQPKKLHRAKSAERRYQQRSTENPQILMMVYSNISKKEKLSGTATLLQDAAHSKYLAVMPGIQTIANSRRHRRSHIFNEHIMGMKHVPSADSSRTLCRRVDFFVDFKQIGWDSWIIHPVKYNAYRCEGECPSPVNERLKPNNHAYMQ

>Q98TU0

MKLWDILATCLLLLSSVSTRPLFHKLQPSKRAVVRSESPALDPIIDSQPETSNPKQASMEEQYDLTGLYPEQFEDVMDFIEATLGRLRRSSDVEPQMKRDRVRQKAAANTEKSGGRGRGERKRSRGRARSRDDRVKGQGRGCLLKEIHLNVTDLDLGYRTKEELIFRYCSGPCHDAETNYDKILNNLTHNKKLDKDTPSRTCCRPIAFDDDISFLDDSLEYHTLKKHSAKKCACV

In this study, we randomly divided 151 surveyed sequences into cross-validation data and independent data for building and testing the models, respectively. We repeated this with process for 10 times when keeping the same sequence number distributions over these two parts. This means that all our experiments were carried out on 10 different datasets. The protein IDs of sequences used in 10 experiments are given below

**Experiment 1

---Cross-validation data ---

O54907; O75888; P32972; P41273; P41274; P50592; Q5UBV8; Q8JFG3; Q8MUJ1; Q9BEA8; Q9ESE2; Q9TSV8; Q9UNG2; Q9WU72; A0S0B0; A6N6I9; A9JSE7; B0R191; C8AW45; H2N2P1; K9M1U5; M0R7X9; O00585; O35793; O43927; O55233; O60542; O61643; O62757; O70460; O88593; O95760; P01583; P01586; P03972; P04351; P05112; P06740; P06744; P09056; P10148; P13232; P15247; P15514; P20808; P21972; P21973; P22004; P22800; P30251; P34819; P35225; P35834; P38440; P41693; P42706; P43021; P43026; P43490; P50228; P51670; P52460; P53347; P55107; P56830; P58499; P81013; P81530; P83714; P91699; Q08782; Q13007; Q1RMP9; Q27913; Q29RT9; Q4PR21; Q60480; Q61728; Q6DF53; Q6ZMJ4; Q80XG2; Q86WN2; Q8IU54; Q8IZ96; Q8NEV9; Q8QFQ8; Q8TAZ6; Q8UUJ9; Q90XB8; Q91Z84; Q920D7; Q96DZ9; Q96FZ5; Q98TU0; Q99731; Q99LJ5; Q9D6G9; Q9DAR1; Q9DAS1; Q9GZX6; Q9H293; Q9H2A7; Q9JI24; Q9JIL2; Q9JLA2; Q9MZR1; Q9NZH7; Q9NZH8; Q9P0M4; Q9QXT6; Q9QYY1; Q9QZM3; Q9WUQ5; Q9WVL7; Q9XT91; Q9YGD3; Q9Z1X0;

--- Independent text data ---

O55237; Q7TS55; Q9JM10; Q9Z2P3; B0ZE70; B5B3U4; O15467; O95715; P02775; P08721; P09920; P18340; P28797; P30034; P30253; P30255; P30782; P40226; P48411; P48969; P55101; P55106; P82535; Q6AYE8; Q8R460; Q8TAD2; Q90YI0; Q9D6Z6; Q9DAC0; Q9HBE4; Q9NPH9; Q9NZH6; Q9TTB0; Q9VAK8;

**Experiment 2

---Cross-validation data ---

O54907; O55237; O75888; P32972; P41273; P50592; Q5UBV8; Q7TS55; Q8JFG3; Q9ESE2; Q9JM10; Q9TSV8; Q9UNG2; Q9WU72; A0S0B0; A9JSE7; B5B3U4; H2N2P1; K9M1U5; M0R7X9; O00585; O43927; O55233; O60542; O61643; O62757; O70460; O88593; O95715; O95760; P01583; P01586; P02775; P04351; P05112; P06740; P06744; P08721; P09920; P10148; P13232; P15247; P15514; P18340; P20808; P21972; P28797; P30034; P30251; P30255; P34819; P35225; P35834; P38440; P40226; P41693; P42706; P43490; P48411; P48969; P50228; P51670; P52460; P53347; P55101; P55106; P55107; P56830; P81530; P82535; P91699; Q08782; Q13007; Q1RMP9; Q27913; Q4PR21; Q60480; Q6AYE8; Q6DF53; Q6ZMJ4; Q80XG2; Q86WN2; Q8IZ96; Q8NEV9; Q8QFQ8; Q8R460; Q8TAD2; Q8TAZ6; Q8UUJ9; Q90XB8; Q90YI0; Q920D7; Q96DZ9; Q98TU0; Q99731; Q9D6G9; Q9D6Z6; Q9DAC0; Q9DAR1; Q9DAS1; Q9GZX6; Q9H293; Q9H2A7; Q9HBE4; Q9JI24; Q9JIL2; Q9JLA2; Q9MZR1; Q9NPH9; Q9NZH8; Q9QXT6; Q9QYY1; Q9TTB0; Q9VAK8; Q9WUQ5; Q9YGD3; Q9Z1X0;

--- Independent text data ---

P41274; Q8MUJ1; Q9BEA8; Q9Z2P3; A6N6I9; B0R191; B0ZE70; C8AW45; O15467; O35793; P03972; P09056; P21973; P22004; P22800; P30253; P30782; P43021; P43026; P58499; P81013; P83714; Q29RT9; Q61728; Q8IU54; Q91Z84; Q96FZ5; Q99LJ5; Q9NZH6; Q9NZH7; Q9P0M4; Q9QZM3; Q9WVL7; Q9XT91;

**Experiment 3

---Cross-validation data ---

O54907; O75888; P32972; P41273; P41274; P50592; Q5UBV8; Q7TS55; Q9BEA8; Q9JM10; Q9TSV8; Q9UNG2; Q9WU72; Q9Z2P3; A0S0B0; A6N6I9; B0R191; B5B3U4; H2N2P1; M0R7X9; O00585; O15467; O35793; O43927; O55233; O61643; O62757; O70460; O88593; O95715; O95760; P01583; P02775; P03972; P04351; P05112; P06740; P06744; P08721; P09056; P09920; P13232; P15514; P18340; P20808; P22004; P22800; P28797; P30034; P30253; P30255; P30782; P34819; P35225; P35834; P38440; P40226; P41693; P43021; P43026; P48411; P50228; P51670; P52460; P53347; P55101; P55106; P56830; P81013; P81530; P82535; P91699; Q08782; Q13007; Q1RMP9; Q29RT9; Q60480; Q61728; Q6AYE8; Q6DF53; Q6ZMJ4; Q80XG2; Q86WN2; Q8IU54; Q8IZ96; Q8NEV9; Q8QFQ8; Q8TAD2; Q8UUJ9; Q90XB8; Q90YI0; Q91Z84; Q920D7; Q96DZ9; Q99731; Q99LJ5; Q9D6G9; Q9D6Z6; Q9DAR1; Q9H2A7; Q9HBE4; Q9JI24; Q9JIL2; Q9JLA2; Q9NPH9; Q9NZH6; Q9NZH7; Q9NZH8; Q9P0M4; Q9QZM3; Q9TTB0; Q9VAK8; Q9WUQ5; Q9WVL7; Q9XT91; Q9YGD3; Q9Z1X0;

--- Independent text data ---

O55237; Q8JFG3; Q8MUJ1; Q9ESE2; A9JSE7; B0ZE70; C8AW45; K9M1U5; O60542; P01586; P10148; P15247; P21972; P21973; P30251; P42706; P43490; P48969; P55107; P58499; P83714; Q27913; Q4PR21; Q8R460; Q8TAZ6; Q96FZ5; Q98TU0; Q9DAC0; Q9DAS1; Q9GZX6; Q9H293; Q9MZR1; Q9QXT6; Q9QYY1;

**Experiment 4

---Cross-validation data ---

O55237; O75888; P32972; P50592; Q5UBV8; Q7TS55; Q8JFG3; Q9BEA8; Q9ESE2; Q9JM10; Q9TSV8; Q9UNG2; Q9WU72; Q9Z2P3; A6N6I9; B0R191; B0ZE70; C8AW45; K9M1U5; M0R7X9; O15467; O35793; O43927; O55233; O60542; O61643; O62757; O70460; O88593; O95715; O95760; P01583; P01586; P02775; P03972; P05112; P06744; P08721; P09056; P09920; P10148; P13232; P15247; P15514; P18340; P20808; P21972; P21973; P22004; P22800; P30251; P30253; P30255; P30782; P35225; P35834; P38440; P40226; P41693; P42706; P43021; P48411; P48969; P50228; P51670; P52460; P53347; P55107; P56830; P58499; P81013; P82535; P83714; P91699; Q08782; Q29RT9; Q4PR21; Q60480; Q61728; Q6AYE8; Q6DF53; Q80XG2; Q86WN2; Q8IU54; Q8IZ96; Q8NEV9; Q8QFQ8; Q8R460; Q8TAD2; Q8TAZ6; Q90XB8; Q90YI0; Q91Z84; Q96FZ5; Q98TU0; Q99731; Q9D6G9; Q9DAC0; Q9DAR1; Q9DAS1; Q9GZX6; Q9H293; Q9HBE4; Q9JIL2; Q9JLA2; Q9NPH9; Q9NZH6; Q9NZH7; Q9NZH8; Q9QXT6; Q9QYY1; Q9TTB0; Q9VAK8; Q9WUQ5; Q9XT91; Q9YGD3; Q9Z1X0;

--- Independent text data ---

O54907; P41273; P41274; Q8MUJ1; A0S0B0; A9JSE7; B5B3U4; H2N2P1; O00585; P04351; P06740; P28797; P30034; P34819; P43026; P43490; P55101; P55106; P81530; Q13007; Q1RMP9; Q27913; Q6ZMJ4; Q8UUJ9; Q920D7; Q96DZ9; Q99LJ5; Q9D6Z6; Q9H2A7; Q9JI24; Q9MZR1; Q9P0M4; Q9QZM3; Q9WVL7;

**Experiment 5

---Cross-validation data ---

O54907; O55237; O75888; P32972; P41274; P50592; Q7TS55; Q8JFG3; Q8MUJ1; Q9BEA8; Q9ESE2; Q9JM10; Q9TSV8; Q9WU72; A0S0B0; A6N6I9; A9JSE7; B0ZE70; B5B3U4; C8AW45; M0R7X9; O00585; O35793; O43927; O62757; O70460; O95715; O95760; P01583; P01586; P02775; P03972; P08721; P09920; P10148; P13232; P15247; P15514; P18340; P20808; P21972; P21973; P22004; P22800; P28797; P30034; P30251; P30255; P30782; P34819; P35225; P35834; P40226; P41693; P42706; P43021; P43490; P48969; P50228; P52460; P53347; P55101; P55106; P55107; P56830; P58499; P81013; P81530; P82535; P83714; P91699; Q08782; Q13007; Q1RMP9; Q27913; Q29RT9; Q4PR21; Q60480; Q61728; Q6AYE8; Q6ZMJ4; Q80XG2; Q86WN2; Q8IU54; Q8IZ96; Q8NEV9; Q8QFQ8; Q8R460; Q8TAD2; Q8UUJ9; Q90XB8; Q90YI0; Q920D7; Q96DZ9; Q98TU0; Q99731; Q9D6G9; Q9D6Z6; Q9DAC0; Q9DAR1; Q9DAS1; Q9GZX6; Q9HBE4; Q9JI24; Q9JLA2; Q9MZR1; Q9NZH7; Q9P0M4; Q9QXT6; Q9QYY1; Q9TTB0; Q9VAK8; Q9WUQ5; Q9WVL7; Q9XT91; Q9YGD3; Q9Z1X0;

--- Independent text data ---

P41273; Q5UBV8; Q9UNG2; Q9Z2P3; B0R191; H2N2P1; K9M1U5; O15467; O55233; O60542; O61643; O88593; P04351; P05112; P06740; P06744; P09056; P30253; P38440; P43026; P48411; P51670; Q6DF53; Q8TAZ6; Q91Z84; Q96FZ5; Q99LJ5; Q9H293; Q9H2A7; Q9JIL2; Q9NPH9; Q9NZH6; Q9NZH8; Q9QZM3;

**Experiment 6

---Cross-validation data ---

O54907; O55237; P32972; P41274; P50592; Q5UBV8; Q7TS55; Q8JFG3; Q8MUJ1; Q9BEA8; Q9ESE2; Q9JM10; Q9TSV8; Q9WU72; A0S0B0; A6N6I9; A9JSE7; B0R191; B0ZE70; B5B3U4; H2N2P1; K9M1U5; O35793; O43927; O55233; O61643; O62757; O95715; O95760; P01583; P01586; P02775; P03972; P04351; P05112; P06744; P08721; P09056; P10148; P13232; P15247; P18340; P21972; P21973; P22004; P22800; P28797; P30034; P30251; P30253; P30255; P34819; P35225; P35834; P38440; P40226; P42706; P43021; P43026; P48411; P50228; P51670; P52460; P53347; P55106; P55107; P56830; P58499; P81013; P91699; Q08782; Q1RMP9; Q27913; Q29RT9; Q4PR21; Q60480; Q61728; Q6AYE8; Q6DF53; Q6ZMJ4; Q80XG2; Q8IU54; Q8QFQ8; Q8TAD2; Q8TAZ6; Q8UUJ9; Q90XB8; Q90YI0; Q91Z84; Q920D7; Q96DZ9; Q96FZ5; Q99731; Q99LJ5; Q9D6G9; Q9D6Z6; Q9DAC0; Q9DAR1; Q9DAS1; Q9GZX6; Q9H293; Q9H2A7; Q9HBE4; Q9JI24; Q9JIL2; Q9JLA2; Q9MZR1; Q9NPH9; Q9NZH7; Q9P0M4; Q9QXT6; Q9QYY1; Q9QZM3; Q9TTB0; Q9WUQ5; Q9XT91; Q9YGD3;

--- Independent text data ---

O75888; P41273; Q9UNG2; Q9Z2P3; C8AW45; M0R7X9; O00585; O15467; O60542; O70460; O88593; P06740; P09920; P15514; P20808; P30782; P41693; P43490; P48969; P55101; P81530; P82535; P83714; Q13007; Q86WN2; Q8IZ96; Q8NEV9; Q8R460; Q98TU0; Q9NZH6; Q9NZH8; Q9VAK8; Q9WVL7; Q9Z1X0;

**Experiment 7

---Cross-validation data ---

O55237; O75888; P41273; P41274; P50592; Q5UBV8; Q7TS55; Q9BEA8; Q9ESE2; Q9JM10; Q9TSV8; Q9UNG2; Q9WU72; Q9Z2P3; A0S0B0; A6N6I9; A9JSE7; B0R191; B0ZE70; B5B3U4; C8AW45; H2N2P1; K9M1U5; M0R7X9; O15467; O35793; O43927; O55233; O60542; O61643; O62757; O70460; O88593; O95715; O95760; P01583; P01586; P03972; P04351; P05112; P06744; P08721; P09056; P09920; P10148; P13232; P15247; P15514; P20808; P21972; P21973; P22004; P22800; P30034; P30782; P34819; P35225; P35834; P38440; P40226; P43021; P43026; P43490; P48411; P50228; P51670; P52460; P53347; P55106; P55107; P58499; P81013; P81530; P83714; Q08782; Q13007; Q1RMP9; Q27913; Q4PR21; Q60480; Q6AYE8; Q6DF53; Q80XG2; Q8IU54; Q8IZ96; Q8NEV9; Q8R460; Q8TAD2; Q8TAZ6; Q8UUJ9; Q90XB8; Q90YI0; Q91Z84; Q96DZ9; Q96FZ5; Q99731; Q9D6G9; Q9D6Z6; Q9DAC0; Q9DAR1; Q9DAS1; Q9H293; Q9H2A7; Q9HBE4; Q9JI24; Q9JLA2; Q9MZR1; Q9NPH9; Q9NZH8; Q9QXT6; Q9QYY1; Q9QZM3; Q9TTB0; Q9VAK8; Q9WUQ5; Q9YGD3; Q9Z1X0;

--- Independent text data ---

O54907; P32972; Q8JFG3; Q8MUJ1; O00585; P02775; P06740; P18340; P28797; P30251; P30253; P30255; P41693; P42706; P48969; P55101; P56830; P82535; P91699; Q29RT9; Q61728; Q6ZMJ4; Q86WN2; Q8QFQ8; Q920D7; Q98TU0; Q99LJ5; Q9GZX6; Q9JIL2; Q9NZH6; Q9NZH7; Q9P0M4; Q9WVL7; Q9XT91;

**Experiment 8

---Cross-validation data ---

O54907; O55237; O75888; P32972; P41273; P50592; Q5UBV8; Q7TS55; Q8JFG3; Q8MUJ1; Q9BEA8; Q9ESE2; Q9UNG2; Q9Z2P3; A0S0B0; A6N6I9; A9JSE7; B5B3U4; C8AW45; H2N2P1; M0R7X9; O00585; O15467; O35793; O43927; O60542; O61643; O62757; O95760; P01583; P01586; P02775; P03972; P05112; P06740; P06744; P09056; P09920; P10148; P13232; P15247; P15514; P18340; P20808; P21972; P21973; P22800; P28797; P30034; P30251; P30253; P30255; P30782; P34819; P35225; P35834; P38440; P40226; P41693; P43021; P43026; P43490; P50228; P51670; P53347; P55101; P55106; P56830; P58499; P83714; P91699; Q08782; Q1RMP9; Q27913; Q29RT9; Q4PR21; Q6AYE8; Q6DF53; Q6ZMJ4; Q80XG2; Q8IU54; Q8IZ96; Q8NEV9; Q8QFQ8; Q8R460; Q8TAD2; Q8UUJ9; Q90YI0; Q91Z84; Q920D7; Q96DZ9; Q96FZ5; Q98TU0; Q99731; Q99LJ5; Q9D6G9; Q9D6Z6; Q9DAC0; Q9DAR1; Q9DAS1; Q9GZX6; Q9JI24; Q9JIL2; Q9JLA2; Q9MZR1; Q9NZH6; Q9NZH7; Q9P0M4; Q9QXT6; Q9QYY1; Q9QZM3; Q9TTB0; Q9VAK8; Q9WUQ5; Q9XT91; Q9YGD3; Q9Z1X0;

--- Independent text data ---

P41274; Q9JM10; Q9TSV8; Q9WU72; B0R191; B0ZE70; K9M1U5; O55233; O70460; O88593; O95715; P04351; P08721; P22004; P42706; P48411; P48969; P52460; P55107; P81013; P81530; P82535; Q13007; Q60480; Q61728; Q86WN2; Q8TAZ6; Q90XB8; Q9H293; Q9H2A7; Q9HBE4; Q9NPH9; Q9NZH8; Q9WVL7;

**Experiment 9

---Cross-validation data ---

O54907; O75888; P41273; P41274; P50592; Q5UBV8; Q7TS55; Q8MUJ1; Q9BEA8; Q9ESE2; Q9TSV8; Q9UNG2; Q9WU72; Q9Z2P3; A0S0B0; A6N6I9; A9JSE7; B0R191; B0ZE70; B5B3U4; H2N2P1; K9M1U5; M0R7X9; O00585; O15467; O43927; O61643; O62757; O88593; O95715; O95760; P01586; P02775; P05112; P06744; P08721; P09056; P09920; P10148; P13232; P15247; P15514; P18340; P20808; P21972; P21973; P22004; P22800; P28797; P30251; P30255; P34819; P35834; P42706; P43026; P43490; P48411; P48969; P50228; P51670; P52460; P53347; P55101; P55106; P55107; P56830; P58499; P81013; P82535; P83714; P91699; Q08782; Q13007; Q1RMP9; Q29RT9; Q61728; Q6AYE8; Q6DF53; Q6ZMJ4; Q80XG2; Q86WN2; Q8IU54; Q8QFQ8; Q8R460; Q8TAD2; Q8TAZ6; Q8UUJ9; Q90XB8; Q90YI0; Q920D7; Q96DZ9; Q96FZ5; Q98TU0; Q99731; Q99LJ5; Q9DAC0; Q9DAR1; Q9DAS1; Q9GZX6; Q9H293; Q9H2A7; Q9JI24; Q9JIL2; Q9JLA2; Q9MZR1; Q9NPH9; Q9NZH6; Q9NZH7; Q9NZH8; Q9P0M4; Q9QXT6; Q9QYY1; Q9QZM3; Q9VAK8; Q9WVL7; Q9XT91; Q9YGD3;

--- Independent text data ---

O55237; P32972; Q8JFG3; Q9JM10; C8AW45; O35793; O55233; O60542; O70460; P01583; P03972; P04351; P06740; P30034; P30253; P30782; P35225; P38440; P40226; P41693; P43021; P81530; Q27913; Q4PR21; Q60480; Q8IZ96; Q8NEV9; Q91Z84; Q9D6G9; Q9D6Z6; Q9HBE4; Q9TTB0; Q9WUQ5; Q9Z1X0;

**Experiment 10

---Cross-validation data ---

O75888; P32972; P41273; P41274; P50592; Q5UBV8; Q7TS55; Q8JFG3; Q9BEA8; Q9ESE2; Q9JM10; Q9UNG2; Q9WU72; Q9Z2P3; A0S0B0; B0R191; B0ZE70; B5B3U4; C8AW45; H2N2P1; M0R7X9; O35793; O55233; O60542; O61643; O62757; O70460; O95715; O95760; P01583; P02775; P03972; P04351; P05112; P06740; P06744; P08721; P09056; P09920; P10148; P15247; P15514; P18340; P20808; P21973; P22800; P30034; P30251; P30253; P30255; P30782; P34819; P35225; P35834; P40226; P41693; P43021; P43490; P48411; P50228; P51670; P52460; P53347; P55101; P55106; P55107; P58499; P81530; P83714; P91699; Q08782; Q13007; Q1RMP9; Q27913; Q29RT9; Q4PR21; Q60480; Q61728; Q6AYE8; Q80XG2; Q86WN2; Q8IU54; Q8IZ96; Q8QFQ8; Q8R460; Q8TAZ6; Q90XB8; Q90YI0; Q920D7; Q96DZ9; Q96FZ5; Q98TU0; Q99731; Q99LJ5; Q9D6G9; Q9D6Z6; Q9DAC0; Q9DAS1; Q9GZX6; Q9H293; Q9H2A7; Q9HBE4; Q9JI24; Q9JIL2; Q9MZR1; Q9NPH9; Q9NZH6; Q9NZH7; Q9NZH8; Q9P0M4; Q9QXT6; Q9QZM3; Q9TTB0; Q9VAK8; Q9WUQ5; Q9YGD3; Q9Z1X0;

--- Independent text data ---

O54907; O55237; Q8MUJ1; Q9TSV8; A6N6I9; A9JSE7; K9M1U5; O00585; O15467; O43927; O88593; P01586; P13232; P21972; P22004; P28797; P38440; P42706; P43026; P48969; P56830; P81013; P82535; Q6DF53; Q6ZMJ4; Q8NEV9; Q8TAD2; Q8UUJ9; Q91Z84; Q9DAR1; Q9JLA2; Q9QYY1; Q9WVL7; Q9XT91;
